# An intelligent workflow for sub-nanoscale 3D reconstruction of intact synapses from serial section electron tomography

**DOI:** 10.1186/s12915-023-01696-x

**Published:** 2023-09-25

**Authors:** Sheng Chang, Linlin Li, Bei Hong, Jing Liu, Yuxuan Xu, Keliang Pang, Lina Zhang, Hua Han, Xi Chen

**Affiliations:** 1 Institute of Automation, Chinese Academy of Sciences, 100190 Beijing, China; 2 School of Artificial Intelligence, University of Chinese Academy of Sciences, 100190 Beijing, China; 3 State Key Laboratory of Multimodal Artificial Intelligence Systems, Institute of Automation, Chinese Academy of Sciences, Beijing, 100190 China; 4 School of Software and Microelectronics, Peking University, 100871 Beijing, China; 5 School of Pharmaceutical Sciences, Tsinghua University, 100084 Beijing, China; 6 School of Future Technology, University of Chinese Academy of Sciences, Beijing, 101408 China

**Keywords:** Serial section electron tomography, 3D EM, Semi-auto locating, Auto-alignment, Missing-information restoration, Semi-auto segmentation, Workflow, Synapse

## Abstract

**Background:**

As an extension of electron tomography (ET), serial section electron tomography (serial section ET) aims to align the tomographic images of multiple thick tissue sections together, to break through the volume limitation of the single section and preserve the sub-nanoscale voxel size. It could be applied to reconstruct the intact synapse, which expands about one micrometer and contains nanoscale vesicles. However, there are several drawbacks of the existing serial section ET methods. First, locating and imaging regions of interest (ROIs) in serial sections during the shooting process is time-consuming. Second, the alignment of ET volumes is difficult due to the missing information caused by section cutting and imaging. Here we report a workflow to simplify the acquisition of ROIs in serial sections, automatically align the volume of serial section ET, and semi-automatically reconstruct the target synaptic structure.

**Results:**

We propose an intelligent workflow to reconstruct the intact synapse with sub-nanometer voxel size. Our workflow includes rapid localization of ROIs in serial sections, automatic alignment, restoration, assembly of serial ET volumes, and semi-automatic target structure segmentation. For the localization and acquisition of ROIs in serial sections, we use affine transformations to calculate their approximate position based on their relative location in orderly placed sections. For the alignment of consecutive ET volumes with significantly distinct appearances, we use multi-scale image feature matching and the elastic with belief propagation (BP-Elastic) algorithm to align them from coarse to fine. For the restoration of the missing information in ET, we first estimate the number of lost images based on the pixel changes of adjacent volumes after alignment. Then, we present a missing information generation network that is appropriate for small-sample of ET volume using pre-training interpolation network and distillation learning. And we use it to generate the missing information to achieve the whole volume reconstruction. For the reconstruction of synaptic ultrastructures, we use a 3D neural network to obtain them quickly. In summary, our workflow can quickly locate and acquire ROIs in serial sections, automatically align, restore, assemble serial sections, and obtain the complete segmentation result of the target structure with minimal manual manipulation. Multiple intact synapses in wild-type rat were reconstructed at a voxel size of 0.664 nm/voxel to demonstrate the effectiveness of our workflow.

**Conclusions:**

Our workflow contributes to obtaining intact synaptic structures at the sub-nanometer scale through serial section ET, which contains rapid ROI locating, automatic alignment, volume reconstruction, and semi-automatic synapse reconstruction. We have open-sourced the relevant code in our workflow, so it is easy to apply it to other labs and obtain complete 3D ultrastructures which size is similar to intact synapses with sub-nanometer voxel size.

**Supplementary Information:**

The online version contains supplementary material available at 10.1186/s12915-023-01696-x.

## Background

Cubic micron scaled subcellular structures and their dynamics play crucial roles in cellular functions. With the sub-nanometer voxel size, researchers can visualize and reconstruct the intricate 3D architecture of cells and organelles, which can help them understand the underlying mechanisms of cellular functions. Richard D. Shoop et al. [1] reconstructed the spine mat on chicken ciliary neurons and found spine morphology is used to control the chemical consequences of synaptic signaling. Gunar Fabig et al. [2] and Ina Lantzsch et al. [3] reconstructed the microtubule in the spindle. They respectively defined novel features that segregate both lagging and paired chromosomes for optimal sperm production and revealed the most prominent drivers of spindle rearrangements are changes in nucleation and catastrophe rate. The complex network of neurons in the brain is responsible for various cognitive processes. Sub-nanometer voxel size volumetric reconstructions can help neuroscientists map out the complex synaptic connections between neurons, providing insights into the neural circuitry that underlies brain function and disease. Jing Liu et al. [4] reconstructed synapses and mitochondria and explore the structural plasticity of synapses and mitochondria in the auditory cortex of mice subjected to fear conditioning. Yun-Tao Liu et al. [5] reconstructed hippocampal synapses and identified type-A GABA receptors in inhibitory synapses. Besides, the structural properties of materials play an essential role in determining their properties and applications. By reconstructing the cubic micron scaled volumes of materials with sub-nanometer voxel size, researchers can gain a deeper understanding of the material’s structure, which can help them design and develop new materials with improved properties and applications. To sum up, it is of utmost importance to reconstruct subcellular structures at the cubic micron scale with sub-nanometer voxel size.

Currently, the primary technique used for revealing the ultrastructure of cellular or subcellular is 3D electron microscopy (EM) [6, 7]. 3D EM methodologies include serial section electron microscopy (SSEM) [8], serial block face scanning electron microscopy (SBEM) [9], focused ion beam scanning electron microscope (FIB-SEM) [10], electron tomography (ET) [11], and serial section electron tomography (serial section ET) [12]. SSEM is to cut the biological tissue into serial ultra-thin sections for microscopic imaging. However, the ultra-thin sections are easily wrinkled and torn during section cutting and collecting. And the resolution of SSEM in the Z-direction is also poorer than that of the imaging plane [13], resulting in the inability to reconstruct ultrastructures such as vesicles in synapses. Both SBEM and FIB-SEM image the surface of the tissue block, which is then removed to reveal the layer beneath [14]. But they use different tools to remove the surface, SBEM uses a diamond knife, and FIB-SEM uses a focused ion beam. Because the surface of the tissue block is imaged, SBEM and FIB-SEM avoid wrinkles and tears. However, their resolution in the Z-direction is still limited by minimum cutting thickness, which is far from the sub-nanometer level. ET images the target from multiple angles by transmission electron microscope (TEM) to obtain a complete reconstruction volume. Based on the tomography imaging methods, ET could achieve the isotropic reconstruction of target structures at the sub-nanometer scale. However, the reconstructed volume of ET is limited by the penetrating ability of transmitted electrons, so it cannot reconstruct complete subcellular structure, such as intact synapses. Serial section ET inherits the sub-nanometer voxel size of ET and could achieve large reconstructed volume similar to SSEM in theory. And because of the thick section, serial section ET is not easy to wrinkle and tear, which is much better than SSEM. Above all, Serial section ET seems the best option for reconstructing complete subcellular structure at the sub-nanometer scale, such as intact synapses.

Based on serial section ET technology, multiple sub-nanometer-scale structures have been reconstructed, such as the spine mat on chicken ciliary neurons [1], the small nodes of Ranvier from mice peripheral nerves [15], and the microtubule in the spindle [2, 3]. However, their process relies heavily on human operation, including ROI localization in serial sections, alignment of serial sections, and reconstruction of target structures, which makes their reconstruction process time-consuming and labor-intensive. Besides, these reconstructed structures are too simple, which are rod-shaped or pie-shaped. So, they do not place high demands on the accuracy and computing time of alignment and segmentation, and manual operations are affordable and can achieve the required results. But their approach is unsuitable for complex subcellular structures, like intact synapses with intertwined structures and tiny vesicles inside.

In order to reconstruct intact synapses, it is necessary to establish an intelligent workflow for sub-nanoscale 3D reconstruction from serial section ET. The workflow mainly needs to tackle the following challenges. The first is the locating ROIs in serial sections. Because ROIs are very small compared to the whole section, and there are always many similar structures in biological samples, it is hard to distinguish the right location in the serial sections. As far as we know, there are no reliable means to assist ROI location. Second, it is not easy to align adjacent section volumes due to various missing information during cutting, imaging and electron tomography. While IMOD [16], Ir-tools [17], TrakEM2 [18, 19], Amira [20], etc., can assist the alignment of sequence volumes, the missing information between the adjacent surfaces of the volumes always makes these methods failed. Third, even if the volumes are aligned, the reconstructed results are incomplete due to the missing information. Compressed sensing [21] could be used to recover the missing information of a single ET volume, but it cannot guarantee the continuity between adjacent volumes. Finally, many works [22–26] have been proposed to segment the target structure in EM volume, the trade-off between automation and high-accuracy is still a compromise issue.

**Fig. 1:**
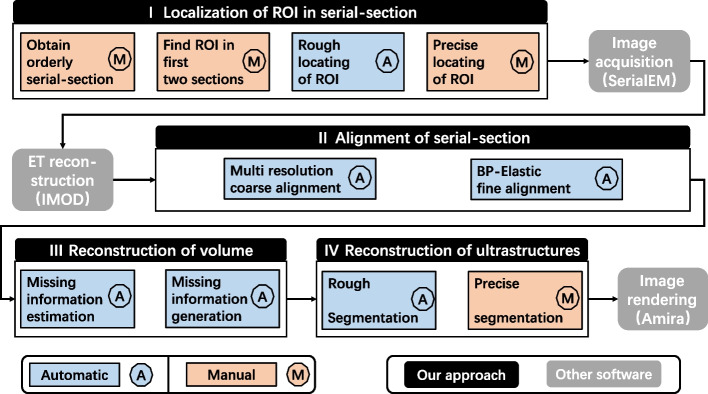
Detail of the workflow. (I) Localization of ROI in serial-section. To locate the ROIs in the serial sections, ordered serial sections are obtained, then affine transformation is used to perform coarse-to-fine ROI localization according to the position relationship between them. (II) Alignment of serial-section. Multi-resolution coarse alignment and BP-Elastic fine alignment of serial sections are performed using the designed algorithm to obtain aligned volumes. (III) Reconstruction of volume. To obtain the intact reconstructed volume, using the designed methods to estimate and generate the missing information of all sections. (IV) Reconstruction of ultrastructures. A 3D neural network combined with manual proofreading was applied to segment synapse ultrastructures. SerialEM, IMOD, and Amira are used for image acquisition, ET reconstruction, and image rendering

In response to the above challenges, we developed an intelligent workflow for sub-nanoscale 3D reconstruction of the intact synapse from serial section ET. First, affine transformations were employed to determine the ROI locations among serial sections based on the relative location of ROIs in sections, which can save time in distinguishing the right location of ROIs. Second, multi-resolution images obtained during the workflow were used to perform multi-scale, coarse-to-fine alignment. Third, the interpolation method based on deep learning generated the missing information between the adjacent surfaces of the volumes to achieve volume reconstruction. Finally, a 3D neural network combined with manual proofreading was applied to segment the membrane and vesicle in synapse with quality and quantity guaranteed.

Our workflow is designed to reconstruct intact synapses at the sub-nanometer scale, but it can also be used to reconstruct other subcellular structures. And our workflow is easy to be applied in other laboratories. Compared to the current mainstream serial section ET workflow, our workflow is faster, more efficient and more accurate.

## Results

Here, we first elaborate on every detail of our workflow to demonstrate the feasibility of our workflow. Then, in order to demonstrate the effectiveness of our workflow, we present an intact synapse from the prefrontal cortex of a wild-type rat brain with sub-nanometer voxel size, reconstructed by the workflow. The reconstructed volume is $1.3\times 1.425\times 0.978$ µm^3^ at a voxel size of 0.664 nm/pixel. Finally, we performed a statistical analysis of the position and the morphology of the reconstructed vesicles in the synapse.

**Fig. 2:**
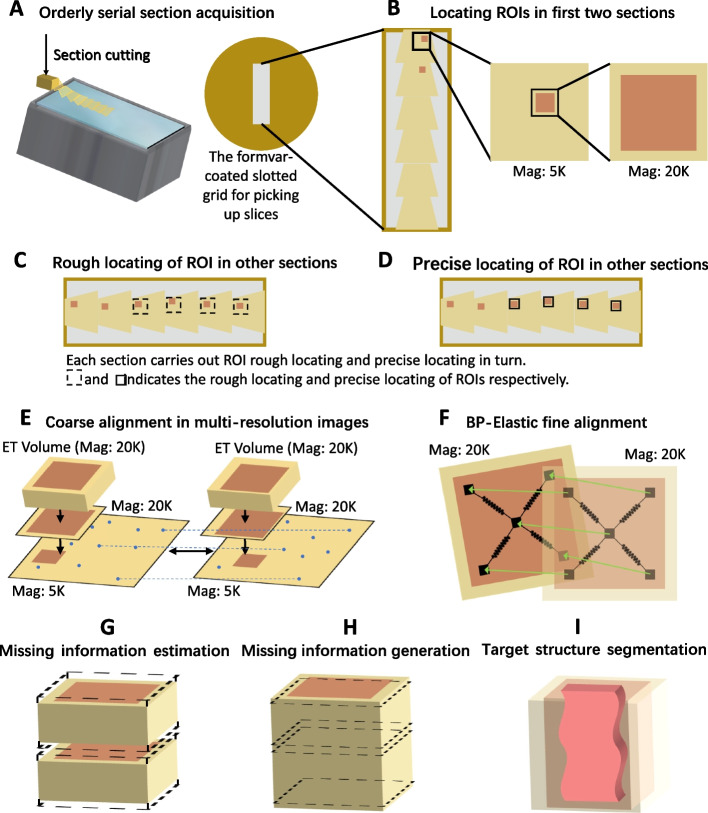
Experimental setup of our workflow. **A** Orderly serial section acquisition. Slice the tissue block with an ultrasonic oscillating diamond knife and collect sections orderly on a formvar-coated slotted grid. **B** Locating ROIs in first two sections. Locating the ROI in the first two sections manually, and take 5K (magnification is 5000) and 20K (magnification is 20000) images of ROI as the reference for other sections. **C** Rough locating of ROI in other sections. Use affine transformation and the position relationship between ROIs to locate the ROI at low magnification (5K). **D** Precise locating of ROI in other sections. Manually determine the precise location (20K) of ROI based on coarse locating. **E** Coarse alignment in multi-resolution images. Make full use of the multi-resolution images obtained during the workflow, and use the feature point-based approach to align the ET volumes coarsely. **F** BP-Elastic fine alignment. Use BP-Elastic to fine align the high-magnification images, then apply the calculated deformation fields to the deformed ET volumes from E. **G** Missing information estimation. Estimate the number of lost images between the adjacent surfaces of the volumes. **H** Missing information generation. Use a frame interpolation approach based on deep-learning network to generate the missing information. **I** Target structure segmentation. Use a 3D neural network combined with manual proofreading to segment the target structure

### Workflow

Our entire workflow is shown in Figs. 1 and  2 provides the general experimental setup. Our workflow could be divided into four parts: localization of ROI in serial-section, alignment of serial-section, reconstruction of volume, and reconstruction of ultrastructures. We describe each part in detail below.

### Localization of ROI in serial-section

As one of the primary means of observing sub-nanostructures, TEM has high resolution with limited field of view (FOV), making it extremely challenging to locate the target structure among different sections. We employ affine transformations to determine the ROI locations among serial sections based on the relative location of ROIs in sections.

**Fig. 3:**
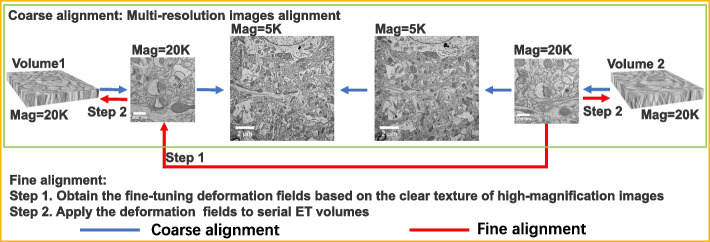
Serial ET volumes alignment diagram. Serial ET volume alignment is mainly divided into two steps. First coarse alignment. It is based on feature matching and affine transformation, and uses multi-resolution images to achieve rough alignment of target structures. Second fine alignment. After the coarse alignment of the volumes, based on the clear texture structure of the high-magnification image, use block matching, BP-Elastic, and thin plate spline to obtain the fine-tuning deformation fields. Then, apply them to serial volumes to correct the alignment of target structures between adjacent volumes

In order to obtain complete reconstruction results and facilitate the locating of subsequent ROIs, it is necessary to ensure that the serial sections are flat and orderly. Therefore, we suggest that the tissue blocks should have a certain hardness and be sliced with a better method to reduce the cutting damage (see the “Sample preparation” section (found under the “Methods” section) for details of our treatment of samples). Then, we placed the sections next to each other on a formvar-coated slotted grid to prevent wrinkles and facilitate observation (as shown in Fig. 2A, B).

For locating and imaging ROIs in serial sections rapidly, it is reasonable to assume that the relative location of ROIs were fixed in serial sections. In TEM, we first locate the adequate candidate positions with the interested structure in one section. Then, we take low-magnification (the magnification is 5000, 5K) and high-magnification (the magnification is 20000, 20K) images at the candidate positions, respectively, which are used to record the approximate position and the accurate position of the interested structure. Next, for a selected candidate position, its validity is further checked in the adjacent section with the help of the low magnification image, and the high magnification image is acquired to record the position in the section (as shown in Fig. 2B). If the selected position is valid, we can use the relative position relationship between the ROIs and the affine transformation to automatically locate ROIs in other sections at low magnification (as shown in Fig. 2C) (for details, please refer to the “ROI rough location” section). And we fine-tune the stage of TEM to record the precise position of ROIs under high magnification (as shown in Fig. 2D). When selected positions in all sections were checked, we locate ROIs of one interested structure. The other candidate positions with the interested structure could be obtained with the similar process repeatedly.

### Alignment of serial-section

Electron tomography (ET) is one of the primary means to observe 3D biological structures at sub-nanometer level. However, due to the inevitable “missing wedge” [27] in electron tomography and tissue lost during cutting [28, 29] and imaging process [30, 31], the adjacent surfaces of ET volumes are different and obscure. These bring great difficulties to align these ET volumes. To solve these problems, we use the multi-resolution images obtained during the imaging process to align the serial ET volumes from coarse to fine. The whole alignment process is shown in Fig. 3.

### Coarse alignment

It is not applicable to align consecutive ET volumes directly for two reasons. First, the inevitable “missing wedge” in ET can cause image obscure at the top and bottom of the volume. Section cutting and irradiation during imaging process also make the reconstructed volume thinner compared to the original volume. These factors are the main sources for the missing information between adjacent volumes. Second, the correspondences between adjacent ET volumes are hard to find because of the limited FOV of volumes. So, we utilize multi-resolution images to perform multi-scale coarse alignment of serial ET volumes.

We exploit the rich content of low-magnification (5k) TEM images to guide the alignment. And the TEM images at the same magnification (20k) of ET volume are used as an intermediate bridge between low-magnification image and ET volume, for the images reconstructed by ET have a great contrast difference with the image directly captured by TEM. Thereby, our workflow realizes automatic coarse alignment of serial ET volumes with sufficient correspondence in every step. The coarse alignment process is shown in Figs. 2E and 3 (for details, please refer to the “Coarse alignment” section).

#### Fine alignment

We use the BP-Elastic algorithm to fine-align the serial ET volumes. Due to the nonlinear deformation of the sample during the slicing and imaging process, which cannot be solved in coarse alignment, the elastic method [32] is used to perform fine alignment. The traditional elastic method relies heavily on the accuracy of block matching. But the surface of the volume reconstructed by ET is obscure, and the high magnification makes the image have many texture-less areas. In addition, the biological samples may also have repetition structures. All of these make it easy to cause mismatches. In response to the above problems, we use the high-magnification TEM images of each section to calculate the deformation fields in elastic, which are used to deform ET volumes. Belief propagation (BP) algorithm [33] is used to correct the mismatch in the block matching process of Elastic. The fine alignment process is shown in Figs. 2F and 3. For the detail of calculation process, see the “Fine alignment” section found under the “Methods” section. The comparison of adjacent images before and after alignment is shown in Additional file 2: Fig. S1.

#### Reconstruction of volume

Due to the missing information during slicing, imaging, and electron tomography, there are still discontinuities in the Z-direction of the reconstructed volume after fine alignment. To solve this problem, we estimate and generate the missing information between the adjacent surfaces of the aligned ET volumes, as shown in Fig. 2G, H.

For simplicity, in our workflow, all missing information is considered as a whole and could be estimated and generated for once. And we assume that the missing image content is uniform along the Z direction. Then, we can estimate and generate the missing information of the reconstructed volume in the Z direction by the pixel difference between adjacent volumes and adjacent slices.

We first estimate the number of lost images between adjacent volumes. The calculation of this number is sophisticated, which is affected by the pixel changes between adjacent volumes and the difference between section thickness in cutting and the thickness of ET volume. All of the volumes are considered simultaneously to make a system of equations (see the “Missing information estimation” section (found under the “Methods” section) for details).

We then generate the missing information between the adjacent surfaces of the aligned ET volumes. A pre-trained network [34] and distillation learning [35, 36] are used to generate the missing information. The pre-trained network has powerful feature extraction and image generation capabilities which are desirable for image interpolation task. Distillation learning can be used to compress the network for small-sample learning. See the “Missing information generation” section (found under the “Methods” section) for details of the network structure, loss function, and training strategy. And see Additional file 3: Fig. S2 for the comparison of volume reconstruction before and after the generation of missing information.

#### Reconstruction of ultrastructures

For the sub-nanoscale reconstruction of ultrastructures in the whole volume, we use a 3D segmentation neural network combined with manual proofreading. Different processing strategies are used for different size of ultrastructures. For presynaptic and postsynaptic membranes, we first use the network to calculate their affinity map and then use the affinity map to obtain the contour of each structure. Then, we modify the contour of target structures manually and use the watershed algorithm to obtain the final segmentation results. For synaptic vesicles, we use the 3D network to obtain the segmentation results directly. Manual modification is also adapted to polish the result. The detail of segmentation methods is shown in the “Target structure segmentation” section.

**Fig. 4:**
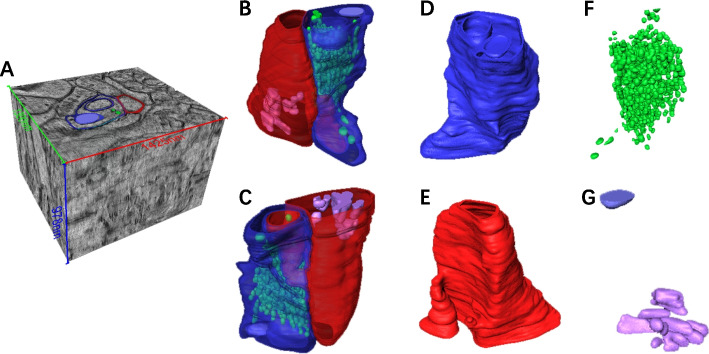
A reconstruction result of a intact synapse. **A** is the image volume which contains the synapse, **B** and **C** are the segmentation results of all structures with different view directions, **D** is the segmented presynaptic membrane, **E** is the segmented postsynaptic membrane, **F** is the segmented vesicle, and **G** are other small structures in the synapse

#### Intact synapse reconstruction

Most synapses are roughly 1 µm^3^ [37] in size, making it impossible for conventional ET to cover the volume entirely. Meanwhile, the diameter of synaptic vesicles is 30 to 40 nm [38], which cannot be reconstructed by SSEM, SBEM and FIB-SEM to reveal their details completely. Therefore, the reconstruction of intact synapses is suitable to show the superiority of our workflow.

Our sample is from the prefrontal cortex of a six-month wild-type rat brain. We stained and embedded the sample and then cut it into 12 consecutive sections with an ultra-sonic oscillating diamond knife. These sections with 100-nm thickness are collected on a formvar-coated slotted grid (see the “Sample preparation” section (found under the “Methods” section) for details). Then, we use JEM-F200 transmission electron microscopy to collect data. The field of view we collected with a magnification of 5K is 10.83 $\times$ 10.83 µm^3^, and the field of view with a magnification of 20K is 2.57 $\times$ 2.57 µm^3^. The voxel size of each sections is close to 45 $\times$ 45 $\times$ 0.1 µm^3^. Five intact synapses among 10 consecutive sections are reconstructed with our workflow finally. One of the reconstructed synapses is shown in Fig. 4. The volume is $1.3\times 1.425\times 0.978$ µm^3^ in size with the voxel size of 0.664 nm/voxel. In Fig. 4, we can clearly see the presynaptic membrane, the postsynaptic membrane, vesicles, and other tiny structures. 

## Discussion

Serial section ET is the best option for reconstructing complete subcellular structures at the sub-nanometer scale. Here, we propose a workflow for sub-nanoscale 3D reconstruction of the intact synapse from serial-section ET. Every step in our workflow is carefully designed and tested, which combines rapid localization of ROIs in serial sections, automatic alignment, restoration, assembly of serial ET volumes, and semi-automatic target structure segmentation. It can significantly improve reconstruction efficiency of serial section ET. And our workflow is open-source and can be easily applied in other laboratories.

Manual localization of ROIs in serial-section is time consuming, which needs to search for ROIs in a large area back and forth in TEM. We employ the affine transformations and the relative position of ROIs in orderly placed sections to determine the rough location of ROI. As a result, our workflow dramatically improves the speed of localization of ROIs in serial-section by narrowing the search area in TEM. Here, the position of collected section on the formvar-coated slotted grid is required to be along a line, which can be achieved easily by expert.

In alignment of serial-section, we take full advantage of the multi-resolution images acquired during the imaging process to align ET volumes from coarse to fine. To tackle texture-less areas and repetition structures in ET volumes, the BP-Elastic algorithm is used to improve alignment accuracy of these positions by utilizing the alignment information of the neighborhood. Thus, the alignment of ET volumes is processed automatically with high accuracy. The parameters of elastic algorithm should be adjusted carefully, which may cause failure of alignment sometimes.

After alignment of ET volumes, we estimate and generate the missing information between adjacent volumes, which is one of the main contributions of our workflow and is not involved in previous serial section ET methods. The pixel changes between adjacent volumes and the difference between section thickness in cutting and the thickness of ET volume are used to estimate the number of images lost for each volume. A pretrained network and distillation learning are used to generate the missing information. Therefore, we can get a more continuous volume along Z-direction.

A 3D segmentation neural network combined with manual proofreading is used to segment the synaptic membranes and vesicles. Because synaptic membranes are next to other membranes closely, minimal missing information may segment them into a whole. Distinguishing different membranes and obtaining their complete contour are the primary missions of synaptic membrane segmentation. We first use the network to calculate affinity maps of different membranes, then modify the map and get the contour of target structures, and finally obtain the segmentation results. Within the presynaptic membrane, synaptic vesicles are distinctly different from other structures, but because they are small, they are prone to wrong and omission during segmentation. We need to verify the segmentation results of the network.

Based on our workflow, we reconstructed five intact synapses with the voxel size of 0.664 nm/voxel. And we analyzed the relationship between the size of synaptic vesicles and the distance between the vesicles and the presynaptic membrane. The presentation and analysis of high voxel size and detailed structure are significant for neuroscience.

## Conclusions

We propose an intelligent workflow for sub-nanoscale 3D reconstruction of intact synapses via serial section ET, which includes semi-automatic ROI localization in serial sections, coarse-to-fine serial ET volume alignment, deep learning-based volume reconstruction, and ultrastructure segmentation. Compared with conventional serial section ET reconstruction methods, our workflow is more automatic and could recover the missing information between adjacent ET volumes. We obtain intact synapses with the proposed workflow and analyze the distance relationship between synaptic vesicles and the active zone. We have open sourced the code of our workflow, which can be implemented easily in other labs. The workflow can be used to obtain complete 3D ultrastructures which size is similar to intact synapses with sub-nanometer voxel size.

## Methods

### Sample preparation

The rat was anesthetized with isoflurane and then perfused with 2$\%$ paraformaldehyde (PFA) and 1.25$\%$ glutaraldehyde (GA) solution via heart. After that, the whole brain sample was removed from the skull and stored in 4$\%$ PFA and 2.5$\%$ GA solution at 4$^{\circ }$C for 12 h. Next, the prefrontal cortex of the brain (1 mm^3^ in size) was post-fixed in phosphate buffer (0.1 M, pH7.4) containing 2$\%$ osmium tetroxide (OsO$_4$) at room temperature for 90 min. For an additional 90 min, swap the staining buffer with 2.5$\%$ ferrocyanide (Sigma) phosphate buffer (0.1 M, pH 7.4), at room temperature. After being rinsed with 0.1 M phosphate buffer three times, the tissues were treated with filtered thiocarbohydrazide (TCH, Sigma) at 40 $^{\circ }$C for 45 min. The tissues were then fixed with 2$\%$ unbuffered 2$\%$ OsO$_4$ solution for 90 min and then incubated with 1$\%$ uranyl acetate (Merck) aqueous solution overnight at 4 $^{\circ }$C. Following a 120-min incubation with a lead aspartate solution (0.033 g lead nitrate (Sigma) in 5 ml of 0.03 M aspartic acid (Sigma), pH 5.0) at 50 $^{\circ }$C, the tissues were dehydrated using a graded ethanol series (50, 70, 80, 90, 100$\%$, 10 min each) and pure acetone. At last, the tissues were embedded by epon-812 resin (SPI). Serial sections (100 nm) were cut with ultra-sonic oscillating diamond knife (DiATOME).

### Imaging equipment and software

We used a JEM-F200 transmission electron microscope and Gatan OneView camera as imaging equipment. During the imaging process, the acceleration voltage is 200 kv. After manually adjusting the focus, brightness, contrast, and astigmatism for each volume, we first recorded the image of the target structure at 5K and then recorded the image of the target structure at 20K. Then, SerialEM was used to acquire Tilt Series images of the target structure between − 60$^{\circ }$ and + 60$^{\circ }$ at 2$^{\circ }$ intervals, and IMOD was used to obtain electron tomography images of the target structure.

### ROI rough location

For the ROI coarse location in ordered serial sections, we first locate the adequate candidate positions of the interested structure in one section; then, we check the candidate structure in the adjacent section. If the structure is indeed what we want, we can use the relative positional relationship between ROIs and the affine transformation matrix to locate the positions of the ROIs in other sections roughly. The position of the ROI on one section can be written as follows:

1 $$\begin{aligned} Loc_n = \left[ \begin{array}{c} x_n \\ y_n \\ 1 \end{array}\right] \end{aligned}$$

The specific formula used to locate the positions of the ROIs in other sections roughly is as follows:

2 $$\begin{aligned} Loc_{n+1}^{rough} = \left\{ \begin{array}{ll} A_1 \times Loc_{n}^{exact}&{} {n = 2} \\ A_2 \times Loc_{n}^{exact}&{} {n = 3} \\ A_n \times Loc_{n}^{exact}&{} {n \ge 4} \end{array}\right. \end{aligned}$$

where $Loc_{n}^{exact}$ is the position of the ROI precisely determined (after rough positioning) in section *n*, $Loc_{n+1}^{rough}$ is the position roughly located in the adjacent section $n+1$, $A_{1}$, $A_{2}$, and $A_{3}$ are the corresponding transformation matrix.

3 $$\begin{aligned} A_1 = \left[ \begin{array}{ccc} 1 &{} 0 &{} tx \\ 0 &{} 1 &{} ty \\ 0 &{} 0 &{} 1 \end{array}\right] \end{aligned}$$

4 $$\begin{aligned} A_2 = \left[ \begin{array}{ccc} s \times cos(\Theta ) &{} -s \times sin(\Theta ) &{} tx \\ s \times sin(\Theta ) &{} s \times cos(\Theta ) &{} ty \\ 0 &{} 0 &{} 1 \end{array}\right] \end{aligned}$$

5 $$\begin{aligned} A_n = \left[ \begin{array}{ccc} a &{} b &{} c \\ d &{} e &{} f \\ 0 &{} 0 &{} 1 \end{array}\right] \end{aligned}$$

These affine matrixes are calculated by exact positions of ROIs in previous sections. The specific ROI rough location calculation process is shown in the Additional file 4: Fig. S3.

### Coarse alignment

For the coarse alignment of serial sections, we utilize multi-resolution and multi-type images obtained during the localization and imaging process. We first scale these images to the same physical pixel-size according to their zoom factor. Then, we extract SIFT feature points [39] of these images and use RANSAC [40] to calculate affine transformation matrix between images to be aligned. The process of coarse alignment can be seen in Fig. 3.

### Fine alignment

For the fine alignment of serial sections, we use elastic model [32] to align the average images of adjacent ET volumes. Because there are texture-less areas and repetition structures in the ET average image (as shown in the Additional file 5: Fig. S4), which may easily cause mismatches, we use the BP model [33] to overcome this difficulty. The specific formula of BP model is as follows:

6 $$\begin{aligned} min_p\left( \sum \limits _{i,j \in V}min \left[ \left\| p_j-p_i-d_i \right\| _2^2, C \right] \right) \end{aligned}$$

where $p_i=(p(x)_i,p(y)_i)$ and $p_j=(p(x)_j,p(y)_j)$ are the centroid coordinates of the $i-th$ and $j-th$ block matching results, respectively, $d_i=(d(x)_i,d(y)_i)$ is the preset distance between $p_i$ and $p_j$, *C* is the truncation threshold used to eliminate the interference of false matching, and *V* represents the set of all matching points. BP model is calculated using an iterative solution similar to gradient descent. The ideal final state for the model is that, for each vertex, the sum of information from all vertices connected to it is minimized.

Then, according to the positions of optimized matching points, we use the thin plate spline (TPS) algorithm [41] to generate deformation fields. Finally, we apply the deformation fields to the corresponding ET volumes to achieve fine alignment.

### Missing information estimation

The main reasons for the missing information are the cutting loss caused by the diamond knife, the missing wedge caused by the insufficient rotation angle, and the shrinkage of sections caused by irradiation. Cutting loss is a direct damage to the surface of the volume. Missing wedges can blur the two ends of the reconstructed volume, thereby affecting the thickness of the final reconstructed volume. We used the “low dose” mode for imaging and the back projection algorithm in IMOD for electronic tomography reconstruction, with the Z factors to correct the shrinkage. However, according to the description in [31], the shrinkage of the sample in the electron microscope can also cause problems similar to “missing wedges,” thereby affecting the thickness of the final reconstructed volume.

The impact of these factors on missing information is complex. To simplify, we consider these factors together and assume that their impact on image content is uniform in the Z direction. Based on this uniform assumption, we can estimate and generate the loss information of the reconstructed volume in the Z direction by the pixel difference between adjacent volumes and adjacent slices.

The estimation of image number accounted for missing information is mainly divided into three steps. In the first step, the number of lost slices in a single volume is estimated based on the difference between section thickness in cutting and the thickness of ET volume. The specific formula is as follows:

7 $$\begin{aligned} L_i^{top} + L_i^{bot} = \frac{L_i^{cut} - L_i^{ET}}{R_{ET}} \end{aligned}$$

where $L_i^{cut}$ and $L_i^{ET}$ represent the thickness in cutting and the thickness of ET volume, respectively, $R_{ET}$ represents the voxel size of the ET reconstructed volume, and $L_i^{top}$ and $L_i^{bot}$ represent the number of lost slices in the top and bottom of the *i*th volumes, respectively, as shown in the Additional file 6: Fig. S5.

In the second step, to make a simple estimation of the number of lost slices between volumes, we exploit the characteristics of the ET reconstructed image, which is that the change of the pixels in sequence images is very slow and can be regarded as piecewise constant. Meanwhile, in order to speed up the calculation, we scaled the image to make its size around $256 \times 256$ and then used the following formula to estimate the number of lost slices between volumes:

8 $$\begin{aligned}{} & {} L_i^{bot}+L_{i+1}^{top} \nonumber \\{} & {} =max(D_{i} \times mean(\mathop {GF}\limits _{(25, 13)}(s_i)+\mathop {GF}\limits _{(25, 13)}(s_{i+1}))) \nonumber \\{} & {} D_{i} = \mathop {GF}\limits _{(10, 3)}(\Vert L_{i+1}^{mean}-L_i^{mean}\Vert ) \end{aligned}$$

where $\mathop {GF}\limits _{(10, 3)}$ and $\mathop {GF}\limits _{(25, 13)}$ represent Gaussian filters with convolution kernel of $10 \times 10$, variance of 3, and convolution kernel of $25 \times 25$, variance of 13. Since the absolute difference of the mean images of adjacent volumes $\Vert L_{i+1}^{mean}-L_i^{mean}\Vert$ may have outliers caused by stain artifact in the images, we use $\mathop {GF}\limits _{(10, 3)}$ to remove the outliers and make $D_{i}$ represent the gray value changes in adjacent volumes. $s_i$ and $s_{i+1}$ are the numbers of images required for each pixel change in the z-direction of volume *i* and volume $i+1$. They are obtained by calculating the number of images needed to change one gray value along z-direction for every pixel in the volume. We use smoothing filter $\mathop {GF}\limits _{(25, 13)}$ to make the correlation of pixel changes smooth in the image.

Finally, based on the calculation results of the previous two steps, the lost slices number of top and bottom of each volume can be calculated using the following formulas.

9 $$\begin{aligned}{} & {} \underset{L_i^{top}, L_i^{bot}}{min}\left( \sum _{i=1}^n \left(\left(L_i^{top} - L_i^{bot}\right)^2\right) + \sum _{i=1}^{n-1} \left(\left(L_{i+1}^{top} - L_i^{bot}\right)^2\right)\right) \nonumber \\{} & {} s.t. \nonumber \\{} & {} \qquad L_i^{top} + L_i^{bot}=c_i^{intra} \nonumber \\{} & {} \qquad L_i^{bot} + L_{i+1}^{top}=c_i^{inter} \end{aligned}$$

where $L_i^{top} + L_i^{bot}=c_i^{intra}$ and $L_i^{bot} + L_{i+1}^{top}=c_i^{inter}$ are the constraints on the loss of images intra and inter volumes respectively, which are constants.

### Missing information generation

Based on the estimation of the missing image number, we generate these images through a deep-learning network. The structure of the network is shown in the Additional file 7: Fig. S6. Since there are not enough training data, the existing networks are prone to overfitting. In order to overcome this problem and lighten the network structure, we use distillation learning [35, 36] to train our network (student network). And because the missing information generation for inter-volume is similar to video interpolation, we select the widely used video interpolation network Super-slomo [34] as the teacher network to guide our network. The loss function of distillation learning is as follows:

10 $$\begin{aligned}{} & {} L=(1-\alpha )L_{rec}^s+\alpha T^2 L_{dis} \nonumber \\{} & {} L_{rec}^s=\Vert \hat{I_i}-I_i \Vert _2 \nonumber \\{} & {} L_{dis}=0.5 \times \Vert N_{flow}^s/T-N_{flow}^t/T \Vert _1 \nonumber \\{} & {} +0.5 \times \Vert N_{arb}^s/T-N_{arb}^t/T \Vert _1 \end{aligned}$$

where $I_i$ represents the real ET image, $\hat{I_i}$ represents the ET image generated by the student network (small network), $N_{flow}^s$, $N_{flow}^t$ represent the output of the optical flow part in the student network and the teacher network respectively, $N_{arb}^s$, $N_{arb}^t$ represent the output of the interpolation part in the student network and the teacher network, respectively, $T=2$ is the temperature in distillation learning, and $\alpha =0.2$ is the weight assigned to different parts that need to be learned in the distillation learning process.

According to the principle of distillation learning, we first use adobe240fps [42], which is used to train the original Super-slomo, to distill the teacher net to obtain a student network with similar feature extraction and fitting ability to the original network. During this process, we used Adam optimizer [43] to train the network for 200 epochs. The learning rate is initialized to 0.0001 and reduced by a factor of 10 at 100 and 150 epochs, respectively. Then, in order to make our student network more suitable for ET data, we selected adjacent ET volume 1 (135 images) and ET volume 2 (106 images) in Fig. 4A from top to bottom to fine-tune the parameters of the network. We choose the middle image of each volume as the fixed image, and the images between the middle images of the two volumes as the dataset to fine-tune the student net, as shown in the Additional file 8: Fig. S7. During the fine-tune training process, the time t corresponding to each image is defined using the serial number of the image as follows.

11 $$\begin{aligned} t=\frac{i}{l_{1}^{ET}/2+M+l_{2}^{ET}/2-1} \end{aligned}$$

where *i* represents the serial number of the image in the fine-tune data, $l_{1}^{ET}$ and $l_{2}^{ET}$ represent the number of images in *Volume*1 and *Volume*2 respectively, and *M* is the total number of images to be generated. We fine-tune the student network for 50 epochs using the Adam optimizer with a learning rate of 0.000001.

**Fig. 5:**
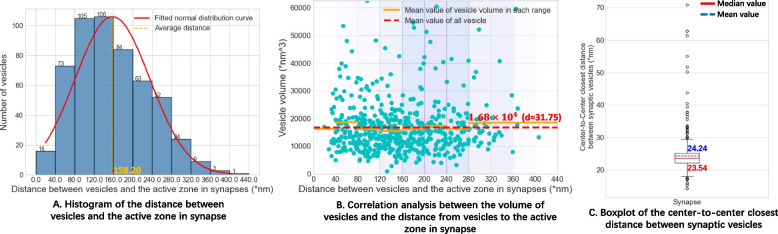
Analysis of reconstructed vesicles. **A** shows the histogram of the distance between synaptic vesicles and the active zone. **B** analyzes the correlation between the volume of vesicles and the distance between vesicles and the active zone. **C** shows the boxplot of the center-to-center closest distance between synaptic vesicles

After fine-tuning, based on the estimated number of lost images, we can use the student network to generate the missing information so as to obtain a whole reconstructed volume.

### Target structure segmentation

Based on the method in [44] and [45], we use U-Net architecture which is implemented in the PyTorch Connectomics library [25] to compose our segmentation network [44]. Since the U-Net [46, 47] structure is widely used for different biological structure segmentation, we use the same network to segment the presynaptic and postsynaptic membranes and vesicles. The loss functions of the network are weightedBCE and Dice. The learning rate adjustment strategy of the network is WarmupCosineLR [48].

Because the synaptic membrane and the synaptic vesicle are very different in size, we use different strategies to segment them. For presynaptic and postsynaptic membranes, we first use the network to calculate their affinity map and then weigh the affinity map and binarize the result to obtain the contour of each structure in the image. Next, we manually modify the contour of target structures and use the watershed algorithm [49] to obtain the segmentation results of different structures. Finally, we select the target structure from these segmentation results and manually correct them. For synaptic vesicles, we use the segmentation result of the presynaptic membrane as the ROI to overcome the imbalanced data caused by the small proportion of vesicles in the whole image. The entire segmentation process is shown in the Additional file 9: Text S2. We first use the network to segment vesicles automatically, then manually modify them to get the final results.

### Supplementary information

**Additional file 1: Text S1.** Explanation of metrics used to evaluate image alignment quality.

**Additional file 2: Figure S1.** Comparison before and after the alignment of adjacent images between adjacent volumes. A is the last image of the first volume in the adjacent volume. B is the first image of the second volume in the adjacent volume. C is the image where A and B are directly superimposed. D is the image where A and B are superimposed after alignment. Ghosting in the red box is much obvious in C. E shows the Peak Signal-to-Noise Ratio (PSNR) values before and after the alignment of adjacent images between ten adjacent volumes. The dashed line is the average PSNR, and the points marked with stars are the PSNR of C and D. The scale bar is 200 nm.

**Additional file 3: Figure S2.** Volume reconstruction results before and after missing information generation. a to f are the volume reconstruction results with copy images. A to F are the volume reconstruction results with generating missing information. a1 to f1 are the volume reconstruction results without generating missing information. a, a1 and A are 3D views of the entire reconstruction volume. b, b1 and B are XY views at the middle of the reconstruction volume. c, c1 and C are XZ views at the middle of the reconstruction volume. d, d1 and D are YZ views at the middle of the reconstruction volume. e, e1 and E, f, f1 and F are enlarged views of the red boxes in c, c1 and C, d, d1 and D, respectively. The comparison between the enlarged images shows that after the generation of missing information, the reconstructed volume is more continuous in the Z direction. G shows the PSNR values before and after the missing information generation of adjacent images between ten adjacent volumes. The dashed line is the average PSNR. The scale bar is 200 nm.

**Additional file 4: Figure S3.** Specific ROI rough location calculation process. It includes the affine transformation matrix and $Loc_{n+1}^{rough}$ calculation process in different stages. Two positions in the same rectangle form a pair.

**Additional file 5: Figure S4.** Texture-less areas and repetition structures in the ET average image of biological samples.

**Additional file 6: Figure S5.** Schematic diagram of the loss of thickness in adjacent volumes.

**Additional file 7: Figure S6.** Missing information generation network. It includes two parts: Teacher Net and Student Net. Each part includes a Flow computation module and an Arbitrary-time flow interpolation module.

**Additional file 8: Figure S7.** Schematic diagram of training and generating data. $I_{1}^{fixed}$ and $I_{2}^{fixed}$ are images in the middle of Volume1 and Volume2, respectively. $I_{i}^{fine-tune}$ is the fine-tune data, *i* is the serial number of the image in fine-tune data set. $l_{1}^{ET}$, $l_{2}^{ET}$ and *M* are the number of images in Volume1, Volume2 and to be generated.

**Additional file 9: Text S2.** Segmentation strategies of synaptic membrane and synaptic vesicle.

**Additional file 10: Figure S8.** More synapse reconstruction results with various sizes and shapes. Voxel size is 0.664*nm*/*pixel*.

**Additional file 11: Text S3.** Evaluation of the generated missing information. The public datasets from [52] and [53] obtained by FIB-SEM and SBF-SEM were used to evaluate the generated missing information. And the method based on image partial phase autocorrelation from [54] was used to quantify the resolution “within” and “across” slices.

**Additional file 12: Text S4.** Comparison of reconstruction results using different methods. The least squares method in TrakEM2 (TrakEM2-ls), Wang et al. proposed method [55] in TrakEM2 (TrakEM2-wang), elastic [56] in TrakEM2 (TrakEM2-elastic), ASAP [52] which used deep learning methods for image alignment, IMOD, and Irtool are used to compare with our workflow.

**Additional file 13: Text S5.** Calculated lost thickness in different synaptic volumes.

**Additional file 14: Movie S1, S2, S3, S4, S5.** Videos of reconstructed intact synapses from serial sections via electron tomography.

## Data Availability

All data generated or analyzed during this study are included in this published article, its supplementary information files, and publicly available repositories. For data, all original electron microscopy data is available via this Science Data Bank repository: https://doi.org/10.57760/sciencedb.06818. All of the data on which the conclusions rely is available via this Figshare repository: https://doi.org/10.6084/m9.figshare.24022485.v1. The source code is available via this Github repository: https://github.com/VictorCSheng/SSET and via the Zenodo public repository with the following doi: https://doi.org/10.5281/zenodo.8275073.

## Appendix

### Analysis of reconstruction results

We first analyze the distance between vesicles and the active zone in synapse, as shown in Fig. 5A. The figure shows the histogram of the distance between synaptic vesicles and the active zone. From the figure, we can see that the distance between synaptic vesicles and the active zone basically conforms to a normal distribution, which is consistent with the statistical results in [50]. And the average distance between synaptic vesicles and the active zone is 158.20 nm. Then, we analyze the correlation between the volume of synaptic vesicles and the distance from synaptic vesicles to the active zone, as shown in Fig. 5B. We grouped the distance between synaptic vesicles and the active zone into different ranges and calculated the average volume of vesicles within the range. From the statistical results in the figure, it can be seen that there is no apparent correlation between the synaptic vesicle volume and the distance from the vesicle to the active zone. The average volume of all synaptic vesicles is $1.68\times10^{4\;}\mathrm{nm}^3$, and the corresponding average diameter is 31.75 nm, which is consistent with the statistical results in [51]. Next, we statistics the center-to-center closest distance between synaptic vesicles, as shown in Fig. 5C. The mean value of the center-to-center closest distance between synaptic vesicles is 24.24 nm.

More synapse reconstruction results are shown in the Additional file 10: Fig. S8. The evaluation of the generated missing information is shown in the Additional file 11: Text S3. The comparison of reconstruction results using different methods is shown in the Additional file 12: Text S4. And the calculated numbers of lost images in different synaptic volumes are shown in the Additional file 13: Text S5. For more displays of the reconstructed intact synapses, please see Additional file 14: Movie S1, S2, S3, S4, S5.

## Abbreviations

EM: Electron microscopy
TEM: Transmission electron microscope
ET: Electron tomography
SSEM: Serial section electron microscopy
SBEM: Serial block face scanning electron microscopy
FIB-SEM: Focused ion beam scanning electron microscope
Serial section ET: Serial section electron tomography
FOV: Field of view
ROIs: Regions of interest
BP: Belief propagation
BP-Elastic: Elastic with belief propagation
TPS: Thin plate spline
PSNR: Peak signal-to-noise ratio
SSIM: Structure similarity index measure
PFA: Paraformaldehyde
GA: Glutaraldehyde
TrakEM2-ls: The least squares method in TrakEM2
TrakEM2-wang: Wang et al. proposed method in TrakEM2
TrakEM2-elastic: Elastic in TrakEM2
