# Supplementary material for: An intelligent workflow for sub-nanoscale 3D reconstruction of intact synapses from serial section electron tomography
Source: BMC Biol. 2023 Sep 25;21:198. doi: 10.1186/s12915-023-01696-x (PMC10519085; doi:10.1186/s12915-023-01696-x)
Supplement: Supplementary file 1 — Additional file 1: Text S1. Explanation of metrics used to evaluate image alignment quality. [file 12915_2023_1696_MOESM1_ESM.pdf]

Supplementary file 1:

Explanation of metrics used to evaluate image alignment quality

The Peak Signal-to-Noise Ratio (PSNR) is calculated based on the Mean Squared Error (MSE) between the reference and aligned images, it reflects the level of fidelity or similarity between the reference and aligned images. A higher PSNR value indicates a higher degree of alignment accuracy. The specific formula of PSNR is as follows:

$$MSE = \frac{1}{n}\sum_{i=1}^{n} (y_i - \hat{y}_i)^2 \tag{1}$$

$$PSNR = 10 \times log_{10}\left(\frac{MAX_I^2}{MSE}\right) \tag{2}$$

where Y and $\hat{Y}$ indicate the reference and aligned image, respectively, y and $\hat{y}$ indicate the pixel value of reference and aligned image, respectively. MAX_I represents the maximum value of image color, with an 8-bit image being 255.

The Structure Similarity Index Measure (SSIM) is another widely used metric for evaluating the quality of image alignment. Unlike PSNR, which only considers pixel-wise intensity differences, SSIM takes into account both structural and textural information in the images, and it is designed to better align with human perception of image quality.) The specific formula of SSIM is as follows:

$$SSIM = \frac{(2\mu_Y \mu_{\hat{Y}} + c_1)(\sigma_{Y\hat{Y}} + c_2)}{(\mu_Y^2 + \mu_{\hat{Y}}^2 + c_1)(\sigma_Y^2 + \sigma_{\hat{Y}}^2 + c_2)} \tag{3}$$

where μ_Y and $\mu_{\hat{Y}}$ indicate the mean pixel value of reference image (Y) and aligned image ($\hat{Y}$), respectively. σ_Y^2 and $\sigma_{\hat{Y}}^2$ indicate the variance of reference image (Y) and aligned image ($\hat{Y}$), respectively. $\sigma_{Y\hat{Y}}$ indicates the covariance of reference image (Y) and aligned image ($\hat{Y}$). c_1 and c_2 are two constants to avoid division by zero.
