# Supplementary material for: An intelligent workflow for sub-nanoscale 3D reconstruction of intact synapses from serial section electron tomography
Source: BMC Biol. 2023 Sep 25;21:198. doi: 10.1186/s12915-023-01696-x (PMC10519085; doi:10.1186/s12915-023-01696-x)
Supplement: Supplementary file 2 — Additional file 2: Figure S1. Comparison before and after the alignment of adjacent images between adjacent volumes. A is the last image of the first volume in the adjacent volume. B is the first image of the second volume in the adjacent volume. C is the image where A and B are directly superimposed. D is the image where A and B are superimposed after alignment. Ghosting in the red box is much obvious in C. E shows the Peak Signal-to-Noise Ratio (PSNR) values before and after the alignment of adjacent images between ten adjacent volumes. The dashed line is the average PSNR, and the points marked with stars are the PSNR of C and D. The scale bar is 200 nm. [file 12915_2023_1696_MOESM2_ESM.pdf]

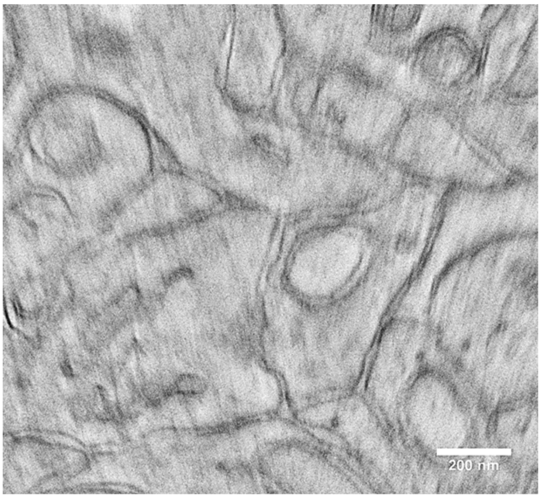

A. The last image of the first volume

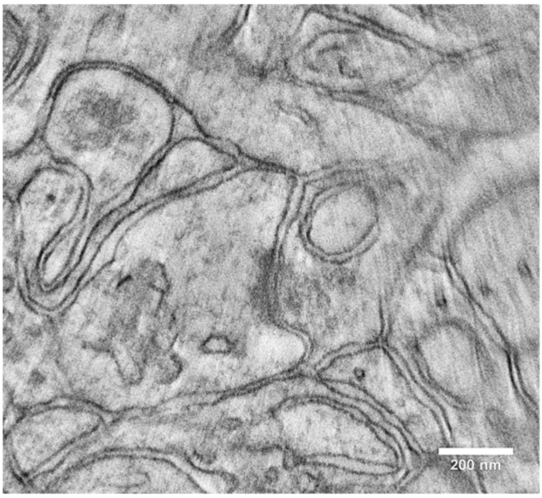

B. The first image of the second volume

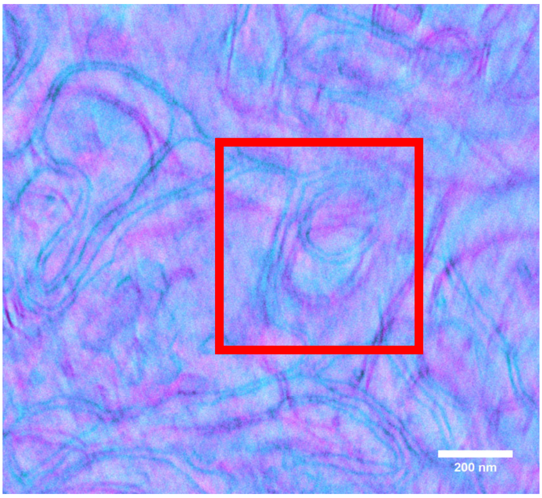

C. Before alignment

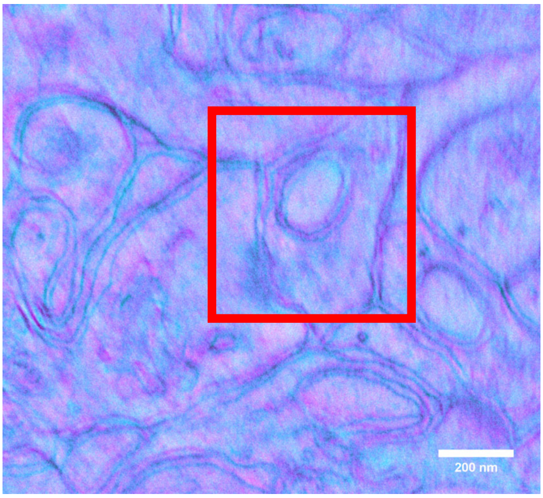

D. After alignment

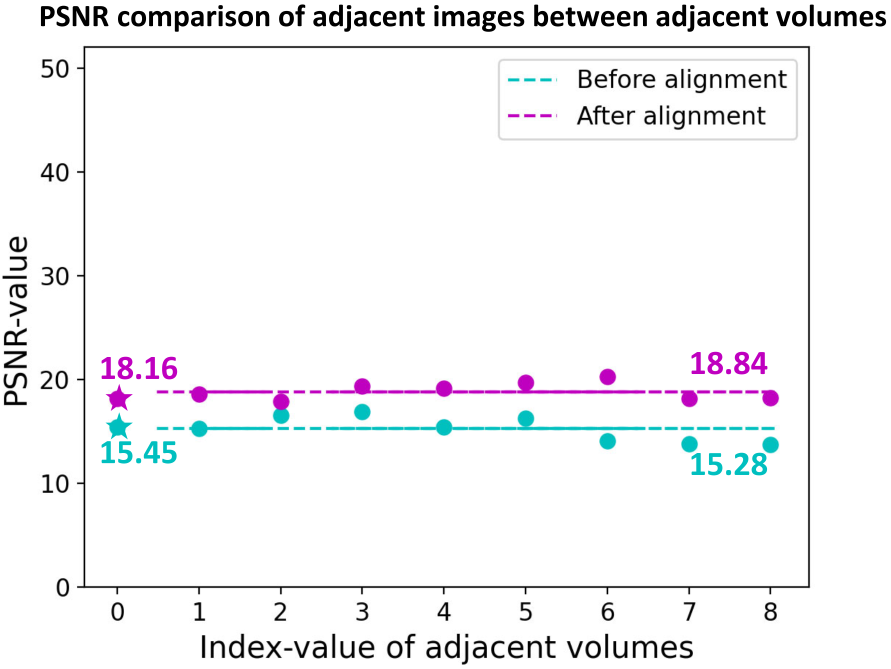

E. Comparison of PSNR between adjacent images before and after alignment
