# Supplementary material for: An intelligent workflow for sub-nanoscale 3D reconstruction of intact synapses from serial section electron tomography
Source: BMC Biol. 2023 Sep 25;21:198. doi: 10.1186/s12915-023-01696-x (PMC10519085; doi:10.1186/s12915-023-01696-x)
Supplement: Supplementary file 3 — Additional file 3: Figure S2. Volume reconstruction results before and after missing information generation. a to f are the volume reconstruction results with copy images. A to F are the volume reconstruction results with generating missing information. a1 to f1 are the volume reconstruction results without generating missing information. a, a1 and A are 3D views of the entire reconstruction volume. b, b1 and B are XY views at the middle of the reconstruction volume. c, c1 and C are XZ views at the middle of the reconstruction volume. d, d1 and D are YZ views at the middle of the reconstruction volume. e, e1 and E, f, f1 and F are enlarged views of the red boxes in c, c1 and C, d, d1 and D, respectively. The comparison between the enlarged images shows that after the generation of missing information, the reconstructed volume is more continuous in the Z direction. G shows the PSNR values before and after the missing information generation of adjacent images between ten adjacent volumes. The dashed line is the average PSNR. The scale bar is 200 nm. [file 12915_2023_1696_MOESM3_ESM.pdf]

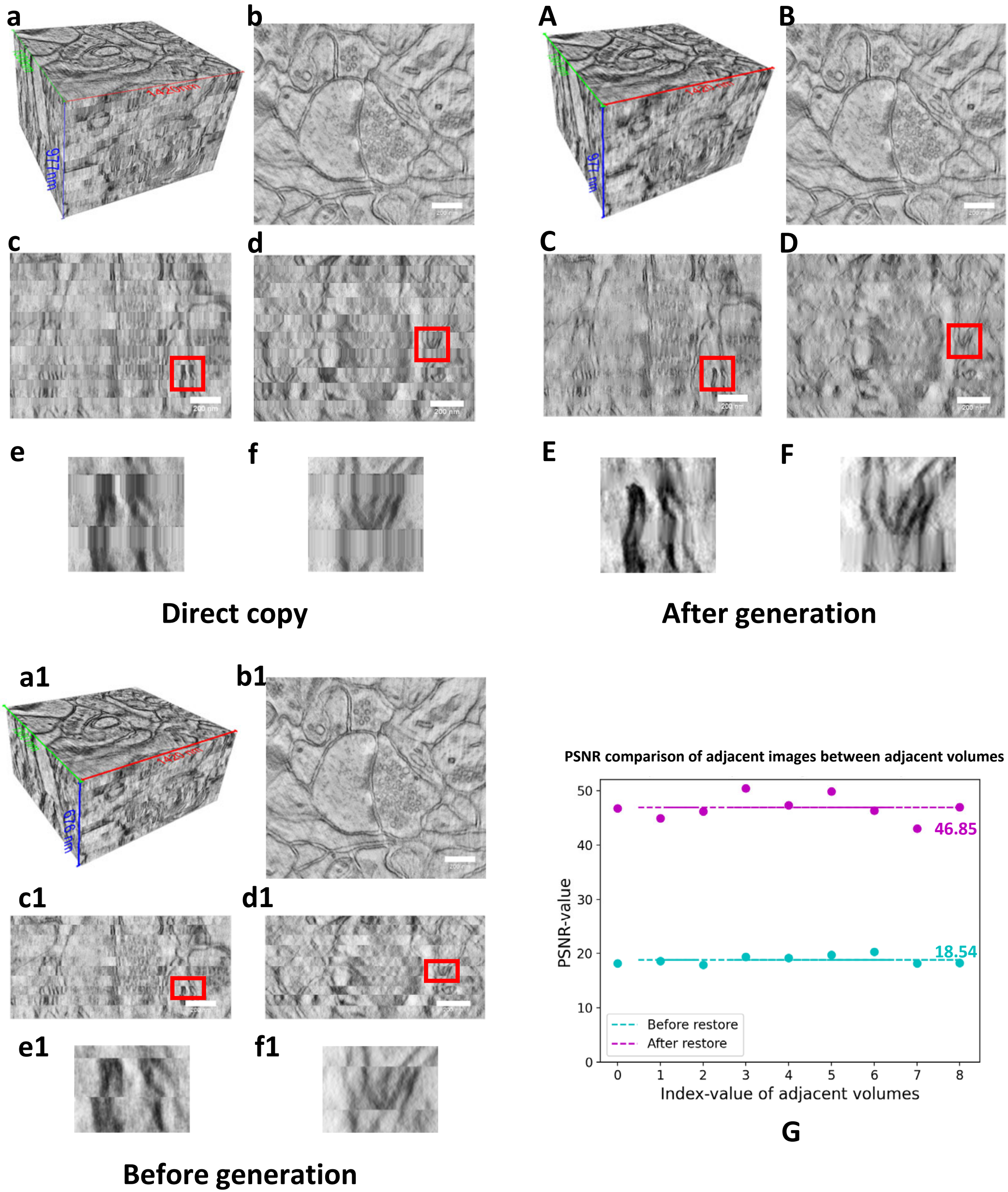

a
b
A
B
1420 nm
977 nm
c
d
C
D
200 nm
e
f
E
F
Direct copy
After generation
a1
b1
c1
d1
e1
f1
1420 nm
976 nm
Before generation
PSNR comparison of adjacent images between adjacent volumes
PSNR-value
Index-value of adjacent volumes
46.85
18.54
Before restore
After restore
0
10
20
30
40
50
0
1
2
3
4
5
6
7
8
G
