# Supplementary figures and images for: An intelligent workflow for sub-nanoscale 3D reconstruction of intact synapses from serial section electron tomography
Source: BMC Biol. 2023 Sep 25;21:198. doi: 10.1186/s12915-023-01696-x (PMC10519085; doi:10.1186/s12915-023-01696-x)

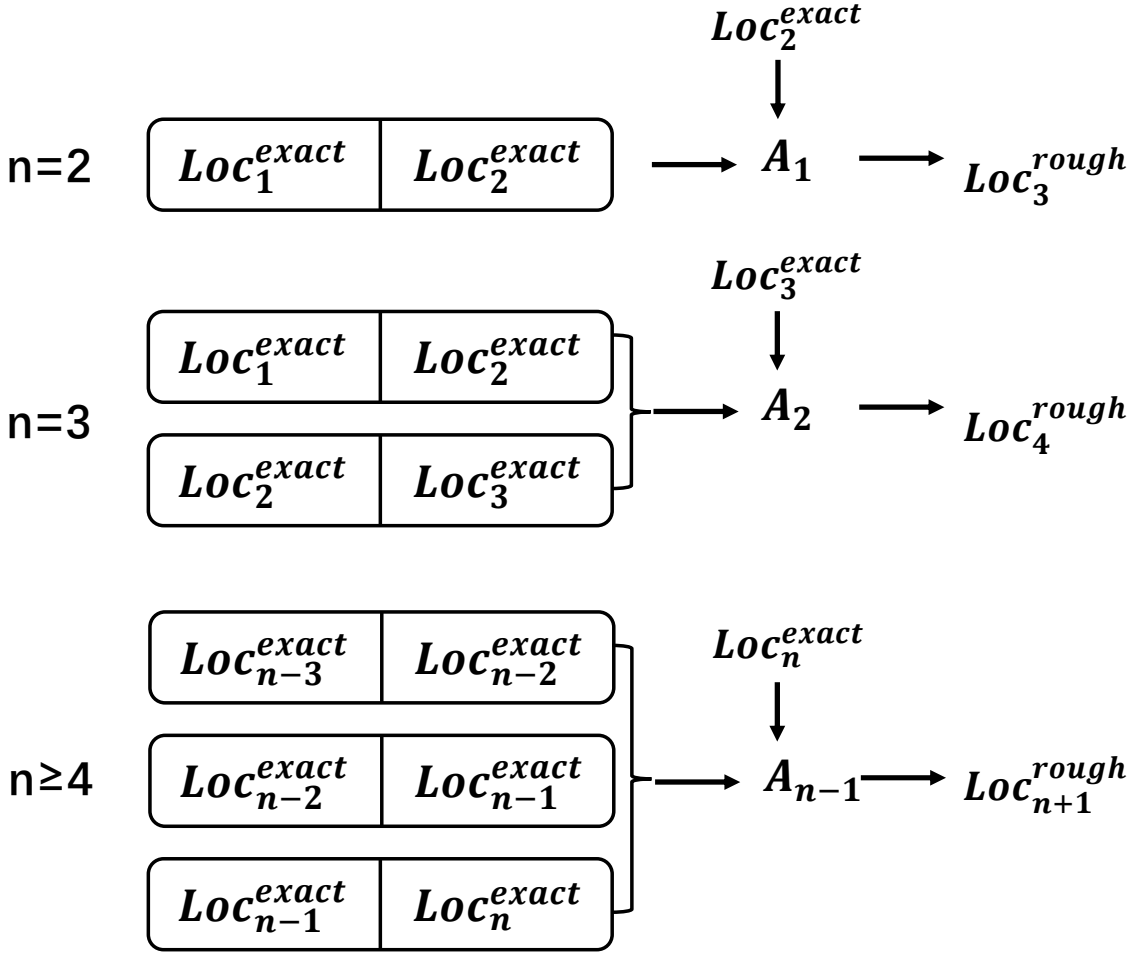

Supplement: Supplementary file 4 — Additional file 4: Figure S3. Specific ROI rough location calculation process. It includes the affine transformation matrix and $Loc_{n+1}^{rough}$Locn+1rough calculation process in different stages. Two positions in the same rectangle form a pair. [file 12915_2023_1696_MOESM4_ESM.pdf]

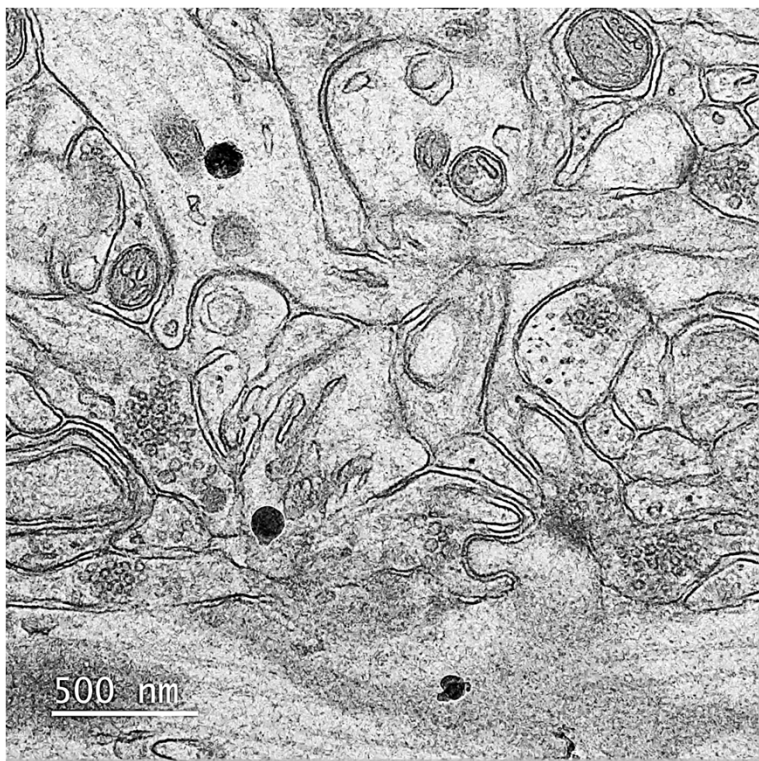

**A**

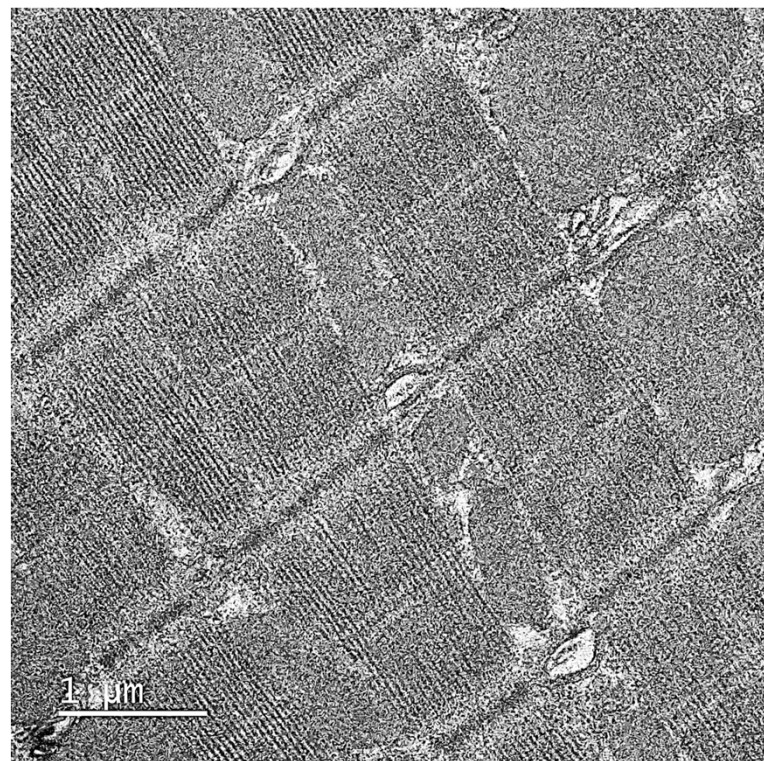

**B**

Supplement: Supplementary file 5 — Additional file 5: Figure S4. Texture-less areas and repetition structures in the ET average image of biological samples. [file 12915_2023_1696_MOESM5_ESM.pdf]

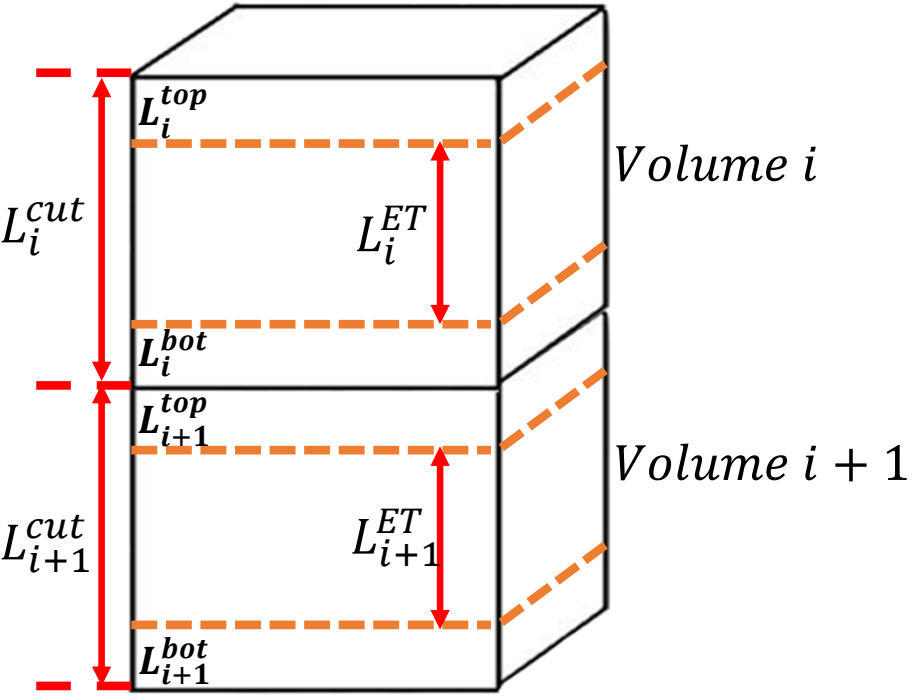

Supplement: Supplementary file 6 — Additional file 6: Figure S5. Schematic diagram of the loss of thickness in adjacent volumes. [file 12915_2023_1696_MOESM6_ESM.pdf]

# Flow computation

# Arbitrary-time flow interpolation

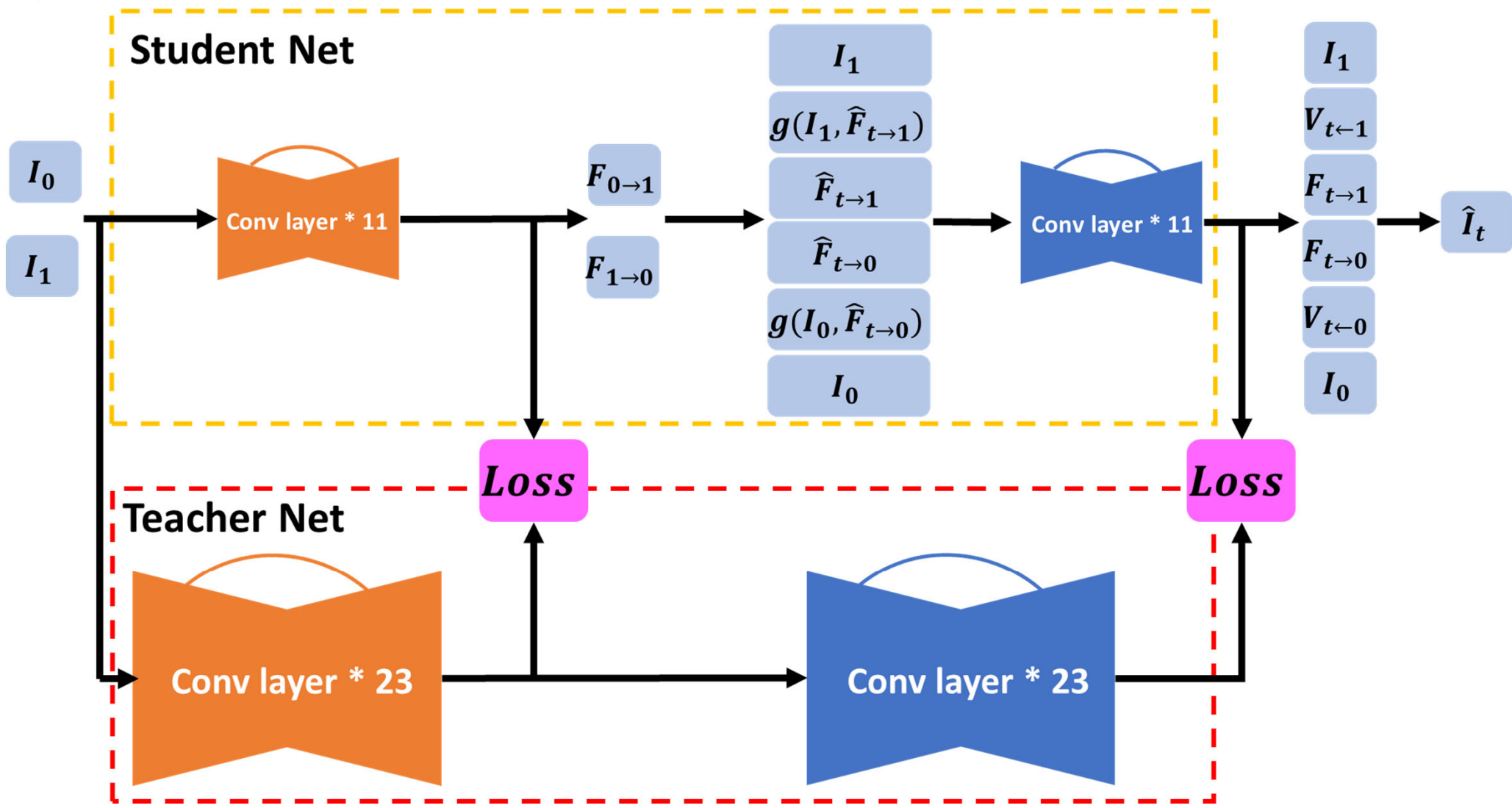

Supplement: Supplementary file 7 — Additional file 7: Figure S6. Missing information generation network. It includes two parts: Teacher Net and Student Net. Each part includes a Flow computation module and an Arbitrary-time flow interpolation module. [file 12915_2023_1696_MOESM7_ESM.pdf]

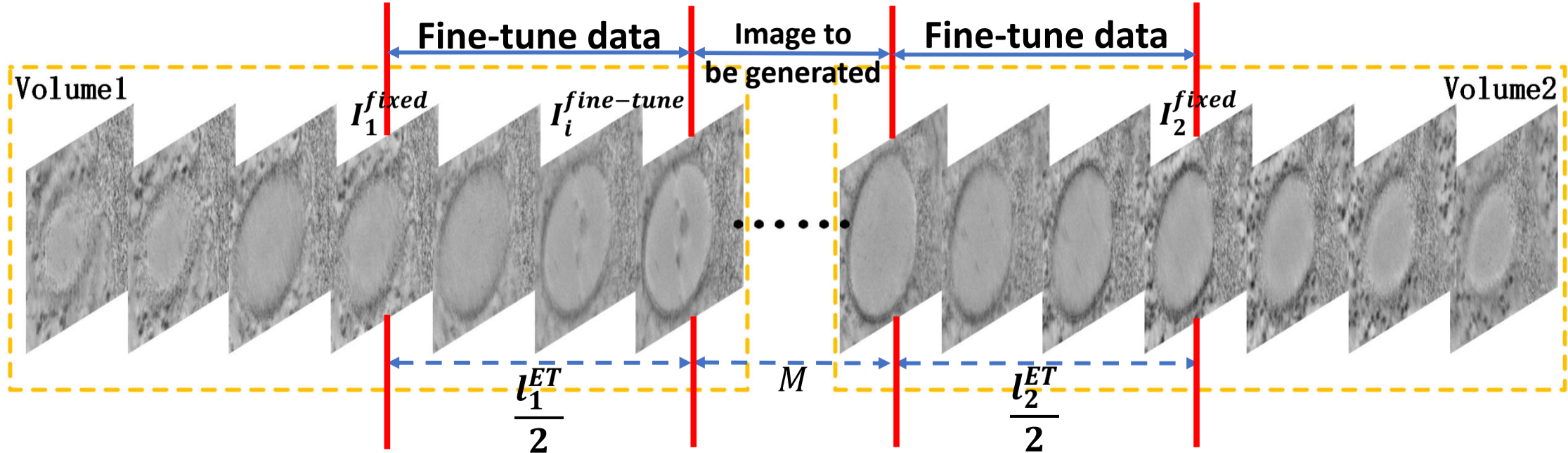

Supplement: Supplementary file 8 — Additional file 8: Figure S7. Schematic diagram of training and generating data. $I_{1}^{fixed}$I1fixed and $I_{2}^{fixed}$I2fixed are images in the middle of Volume1 and Volume2, respectively. $I_{i}^{fine-tune}$Iifine-tune is the fine-tune data, i is the serial number of the image in fine-tune data set. $l_{1}^{ET}$l1ET, $l_{2}^{ET}$l2ET and M are the number of images in Volume1, Volume2 and to be generated. [file 12915_2023_1696_MOESM8_ESM.pdf]

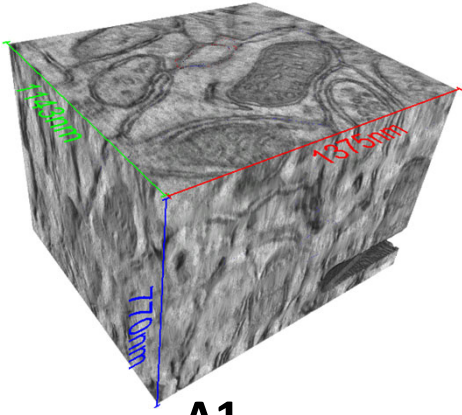

**A1**

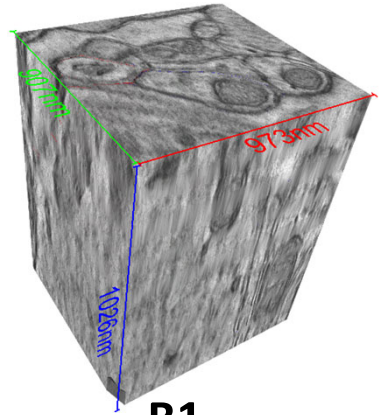

**B1**

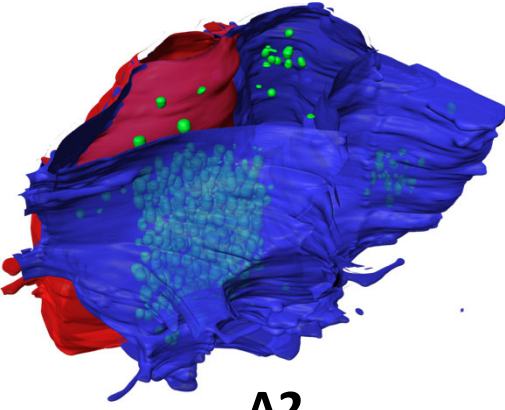

**A2**

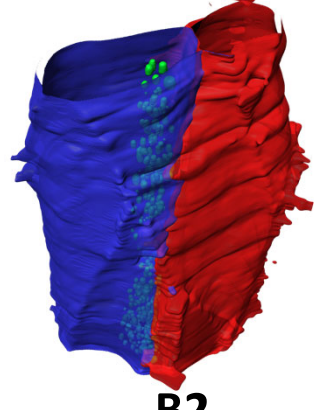

**B2**

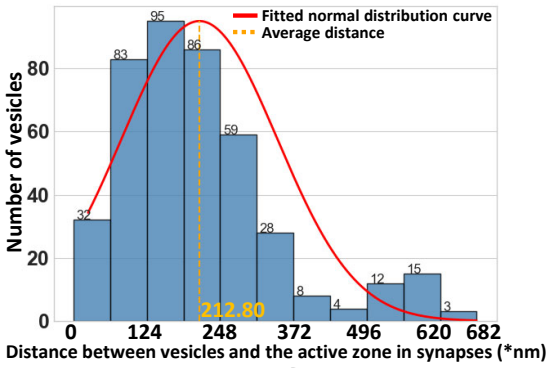

**A3**

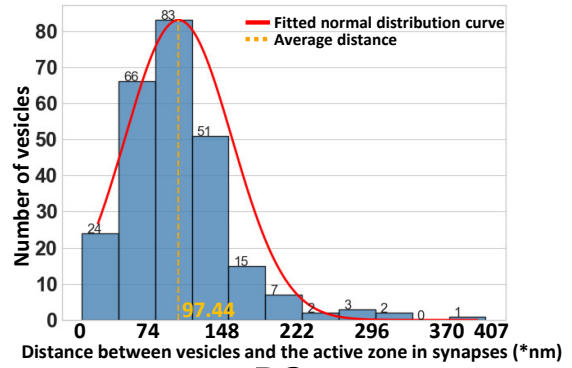

**B3**

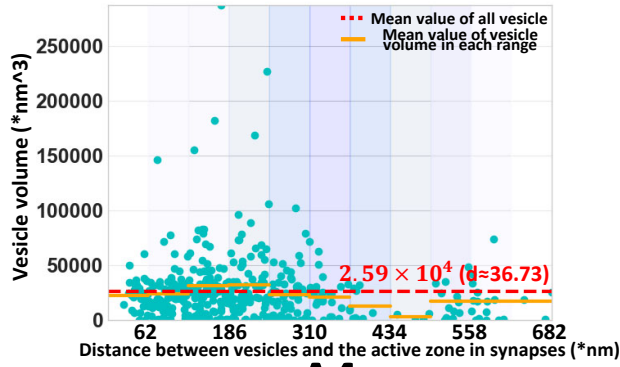

**A4**

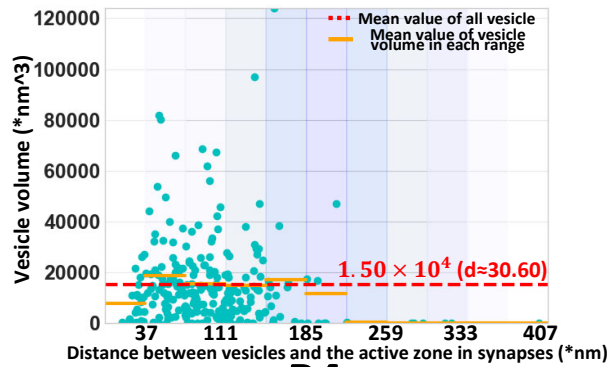

**B4**

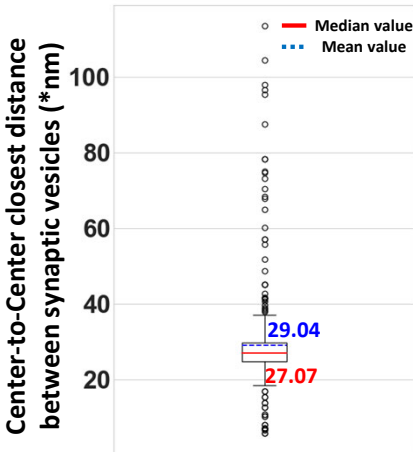

**Synapse A5**

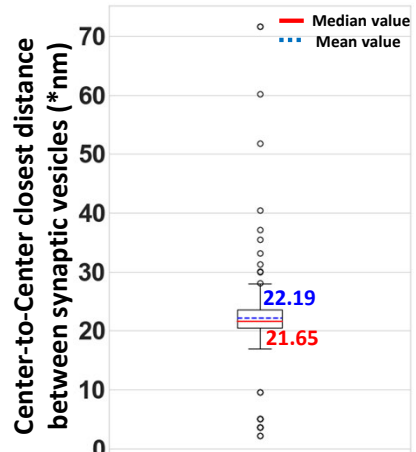

**Synapse B5**

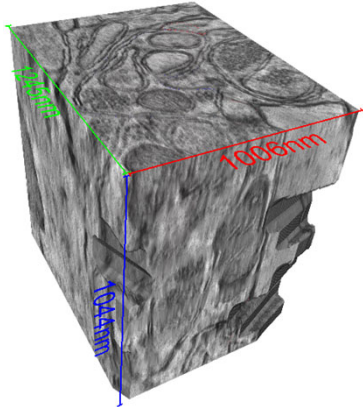

**C1**

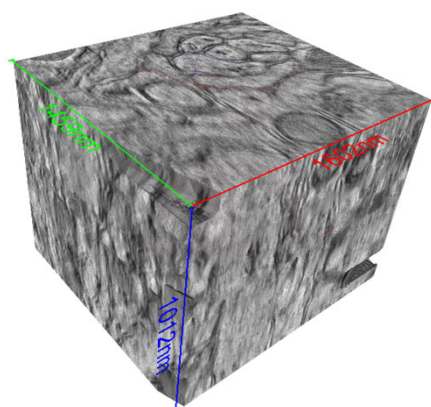

**D1**

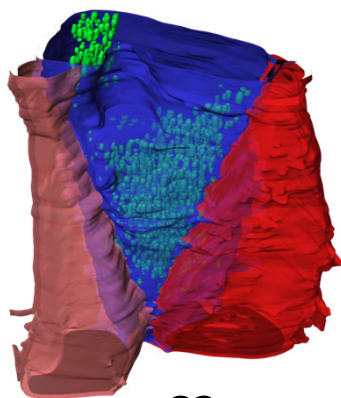

**C2**

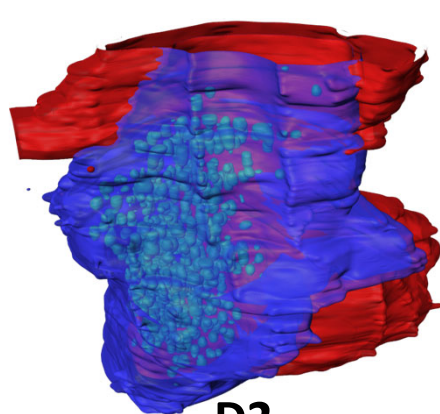

**D2**

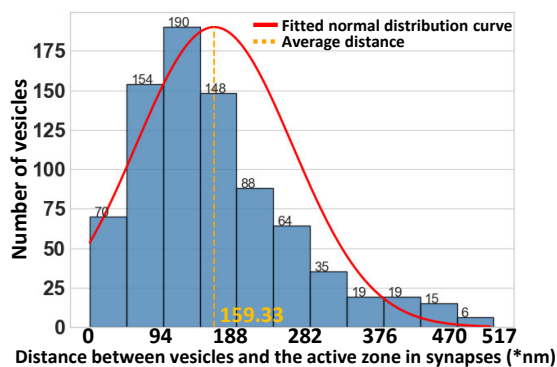

**C3**

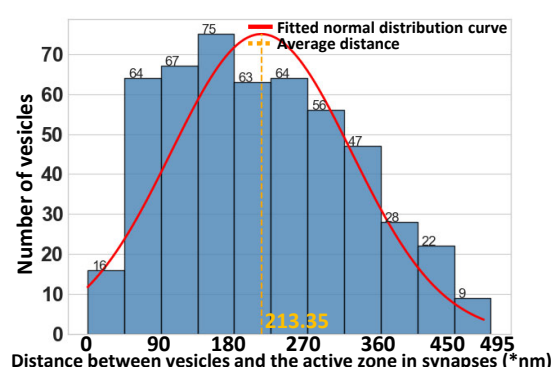

**D3**

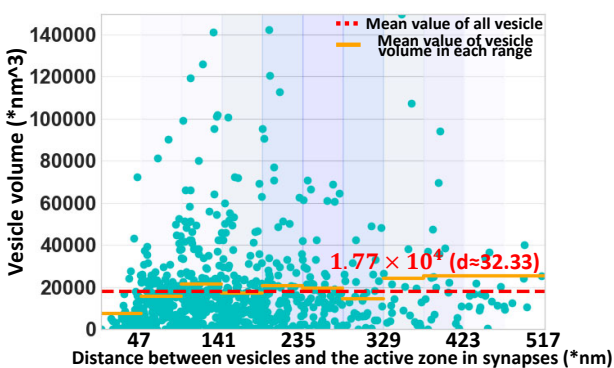

**C4**

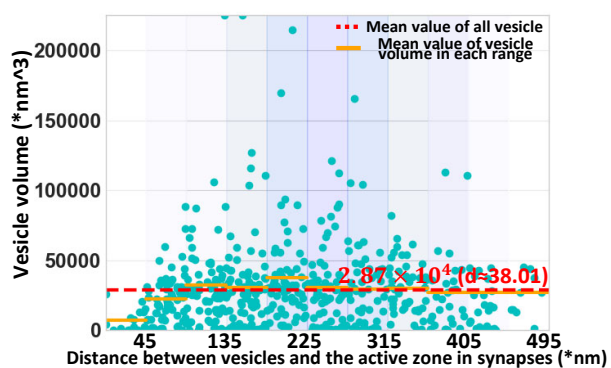

**D4**

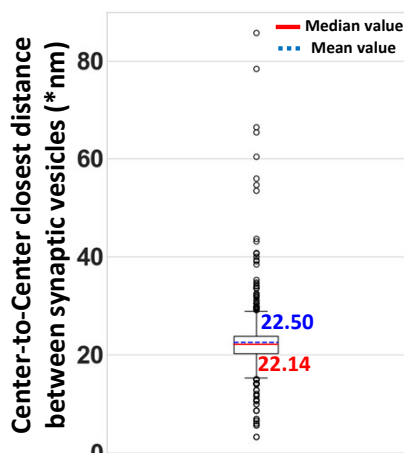

**Synapse C5**

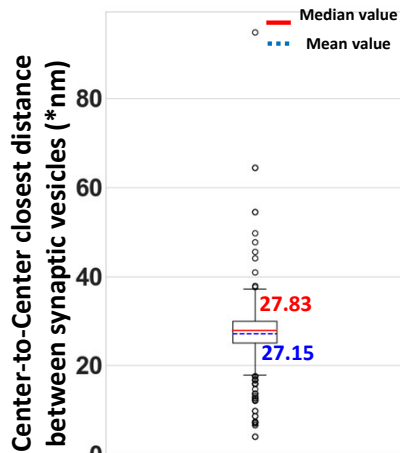

**Synapse D5**

Supplement: Supplementary file 10 — Additional file 10: Figure S8. More synapse reconstruction results with various sizes and shapes. Voxel size is 0.664nm/pixel. [file 12915_2023_1696_MOESM10_ESM.pdf]
