# Supplementary material for: An intelligent workflow for sub-nanoscale 3D reconstruction of intact synapses from serial section electron tomography
Source: BMC Biol. 2023 Sep 25;21:198. doi: 10.1186/s12915-023-01696-x (PMC10519085; doi:10.1186/s12915-023-01696-x)
Supplement: Supplementary file 9 — Additional file 9: Text S2. Segmentation strategies of synaptic membrane and synaptic vesicle. [file 12915_2023_1696_MOESM9_ESM.pdf]

Supplementary file 9:

Segmentation strategies of synaptic membrane and synaptic vesicle

Fig. S1 shows the segmentation process of presynaptic and postsynaptic membranes. Fig. S2 shows the segmentation process of vesicles in the synapse.

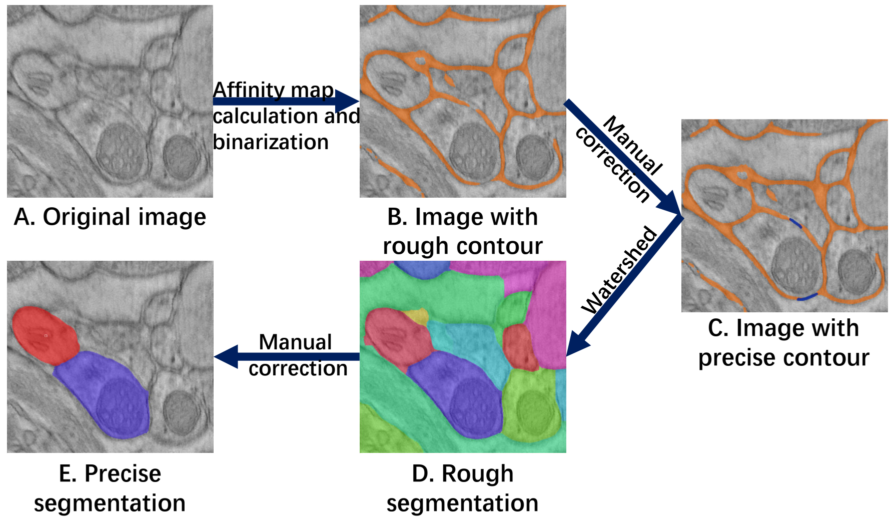

Figure S1 The segmentation process of presynaptic and postsynaptic membranes.

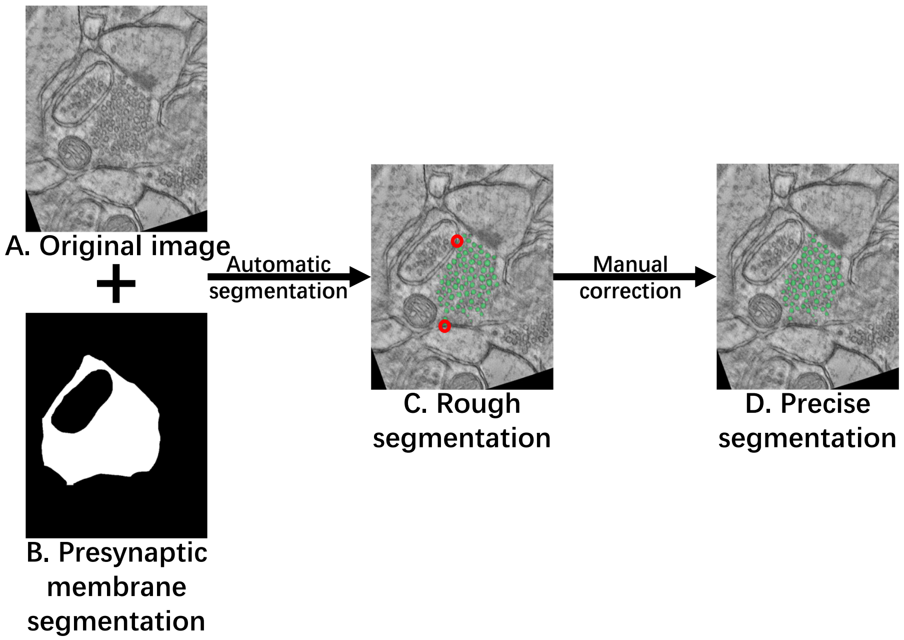

Figure S2 The segmentation process of vesicles in the synapse.

During the training stage, we randomly selected 16 consecutive images, labeled each membrane structure and vesicle as the training set, and used them to fine-tune the network. During the validation stage, we randomly selected 8 consecutive

images and labeled the pre-synaptic and post-synaptic membranes and vesicles as the validation set. Fig. S3 shows one data from the validation set. In the validation set, we obtained an accuracy of 0.99 and a recall of 0.92 for the segmentation of presynaptic and postsynaptic membranes. And for the segmentation of vesicles, we obtained an accuracy of 0.97 and a recall of 0.75. Due to our use of pixel-wise comparison, the accuracy and recall rates obtained for presynaptic and postsynaptic membranes are higher, while the recall rate for vesicles is lower. (The training and validation data has been uploaded to https://github.com/VictorCSheng/SSET and https://doi.org/10.6084/m9.figshare.24022485.v1.)

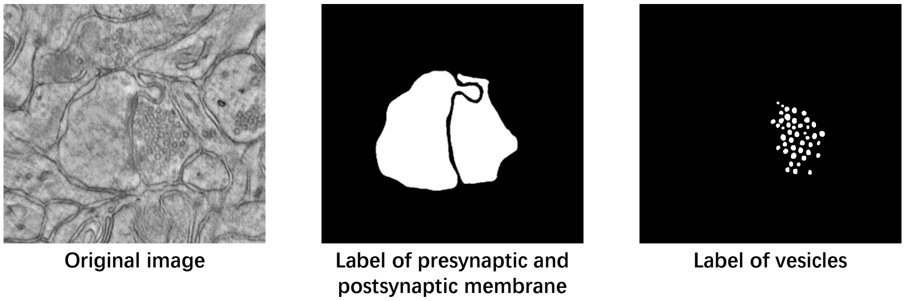

Figure S3 One data from the validation set.
