# Supplementary material for: An intelligent workflow for sub-nanoscale 3D reconstruction of intact synapses from serial section electron tomography
Source: BMC Biol. 2023 Sep 25;21:198. doi: 10.1186/s12915-023-01696-x (PMC10519085; doi:10.1186/s12915-023-01696-x)
Supplement: Supplementary file 11 — Additional file 11: Text S3. Evaluation of the generated missing information. The public datasets from [52] and [53] obtained by FIB-SEM and SBF-SEM were used to evaluate the generated missing information. And the method based on image partial phase autocorrelation from [54] was used to quantify the resolution “within” and “across” slices. [file 12915_2023_1696_MOESM11_ESM.pdf]

Supplementary file 11:

Evaluation of the generated missing information

In order to tackle the discontinuity of the reconstructed volume in the z-direction caused by the missing information during slicing, imaging, and electron tomography, we estimated and generated the missing information between adjacent volumes. Here, we evaluate the generated missing information.

First, the missing information we generate is not created out of zero. Our missing information generation network first learns the optical flow ($F_{0\to1}$ and $F_{1\to0}$) between the known aligned images (I_0 and I_1). It then uses the optical flow to perform various combinations with the known aligned images to generate the missing image $\hat{I}_t$. Fig. S1 shows the input of the network, intermediate calculated optical flow, output of the network, and compares the network output with the ground truth (I_t). It can be seen from the figure that $\hat{I}_t$ which is the estimation of I_t, is calculated by I_0, I_1, $F_{0\to1}$ and $F_{1\to0}$. We can see that the difference between I_t and $\hat{I}_t$ is very small, the Heat map of $\|I_t - \hat{I}_t\|$ is basically black (0), the average of $\|I_t - \hat{I}_t\|$ is only 8.41. The result demonstrated that the image we generate is reasonable.

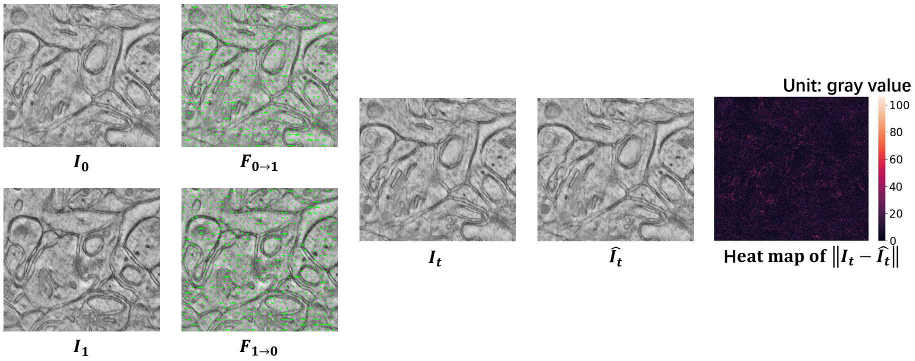

Figure S1 The display of the missing image generation process and the error comparison between the generated image and the actual image. I_0 and I_1 are the middle image of ET volume 9 and ET volume 10 in Fig. S7 from top to bottom. They serve as inputs to the missing information generation network. $F_{0\to1}$ and $F_{1\to0}$ are optical flows from I_0 to I_1 and from I_1 to I_0, respectively. They are the intermediate outputs of the network. I_t is the second image between I_0 and I_1. And $\hat{I}_t$ is the image generated by the network, which is corresponding to I_t. The Heat map of $\|I_t - \hat{I}_t\|$ is basically black (0).

Then we conducted simulation experiments on the public dataset obtained by FIB-SEM imaging technology and SBF-SEM imaging technology to validate the reconstructed volume. The dataset of FIB-SEM represents a $5{\times}5{\times}5\mu m^3$ section taken from the CA1 hippocampus region of the brain, corresponding to a 1065×2048×1536 volume. The resolution of each voxel is approximately $5{\times}5{\times}5nm^3$. It is mainly used to segment mitochondria and synapses. Therefore, the image content is similar to our data. We selected a volume with a clear membrane structure (as shown in Fig. S2(A)), whose size is $1390 \times 1300 \times 500nm^3$, to form a simulation dataset. And the simulated data is divided into different sections with a loss of 30nm (15nm

on the upper and lower surfaces) in 100nm thickness, for missing information estimation and generation.

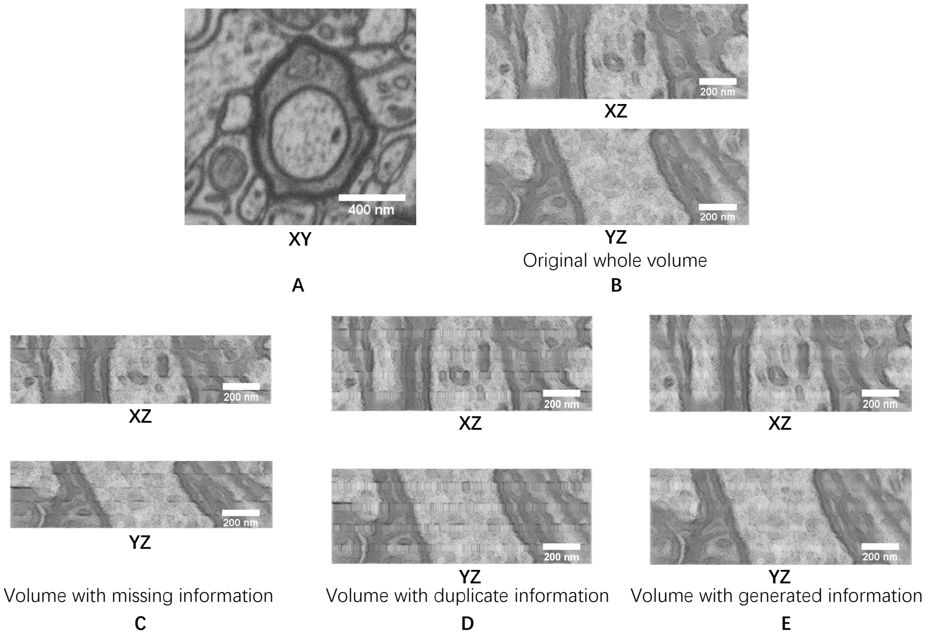

Figure S2 Reconstructed volume validation based on FIB-SEM data.

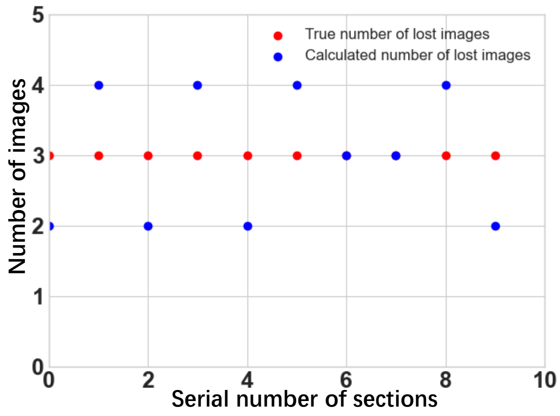

Figure S3 Comparison between the estimated number of lost images and the actual number of lost images on FIB-SEM data.

The dataset of SBF-SEM represents a $61.8 \times 94.8 \times 92.6 \mu m^3$ section taken from the layer 4 of mouse primary somatosensory cortex (P28), corresponding to a $5496 \times 8431 \times 3306$ volume. The resolution of each voxel is approximately $11.24 \times 11.24 \times 28 nm^3$. It is mainly used to reconstruct connectomic in layer 4 of the somatosensory cortex. Therefore, the image content is somewhat similar to our data. We selected a volume with a clear vascular structure (as shown in Fig. S4(A)), whose size is $8722 \times 9441 \times 1500 nm^3$, to form a simulation dataset. And the simulated

data is divided into different sections with a loss of 112nm (56nm on the upper and lower surfaces) in 420nm thickness, for missing information estimation and generation.

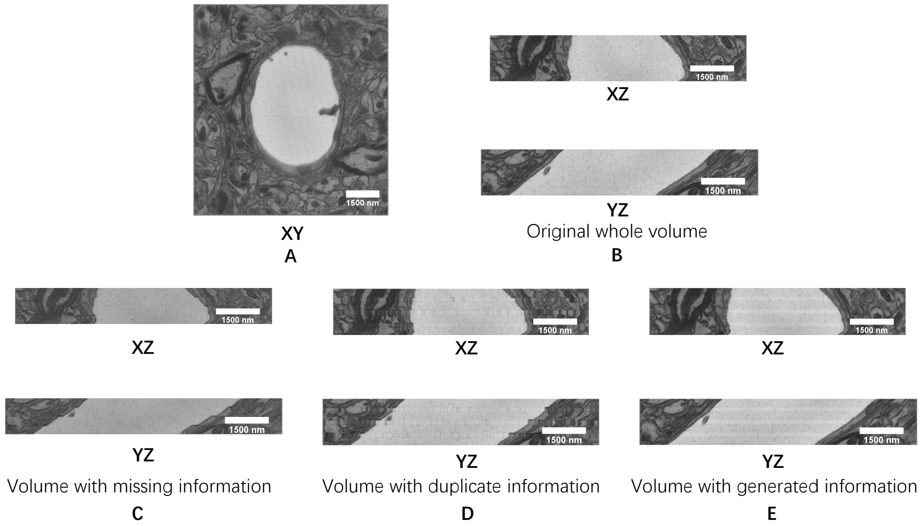

Figure S4 Reconstructed volume validation based on SBF-SEM data.

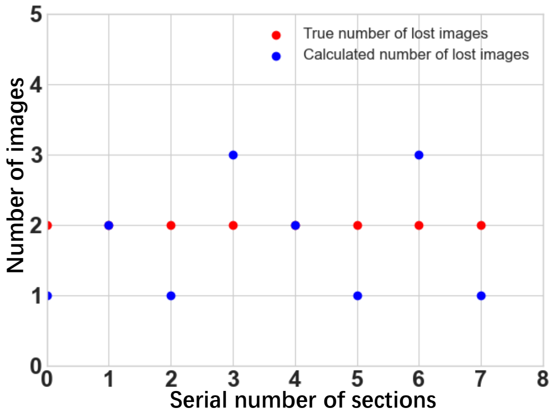

Figure S5 Comparison between the estimated number of lost images and the actual number of lost images on SBF-SEM data.

The comparison between the estimated number of lost images and the actual number of lost images is shown in Fig. S3 and Fig. S5 (for FIB-SEM dataset every lost image corresponding to 5nm, for SBF-SEM dataset every lost image corresponding to 28nm.). It can be seen that our estimation is relatively accurate. On FIB-SEM data, the average difference and the standard deviation between the estimated value and the actual value are 0.8 and 0.4, respectively. On SBF-SEM data, the average difference and the standard deviation between the estimated value and the actual value are 0.75 and 0.43, respectively.

The comparison of the XZ and YZ directions between the generated information reconstructed volume, the duplicate information reconstructed volume, the missing

information reconstructed volume, and the original intact volume are shown in Fig. S2 and Fig. S4. It can be seen that the generated information is closer to the original information, increasing the continuity of the reconstructed volume in the Z direction.

Although there is no actual data in our generated image, we can still ensure the consistency of pixel changes in the volume. We use the adjacent ET volume 9 and ET volume 10 in Fig. S7 from top to bottom. As shown in Fig. S6, in sequence images, the data we generate is very close to the actual data in the part where actual data exists. And in the part where there is no actual data, the changing trend of the data we generate is consistent with the changing trend of the actual data.

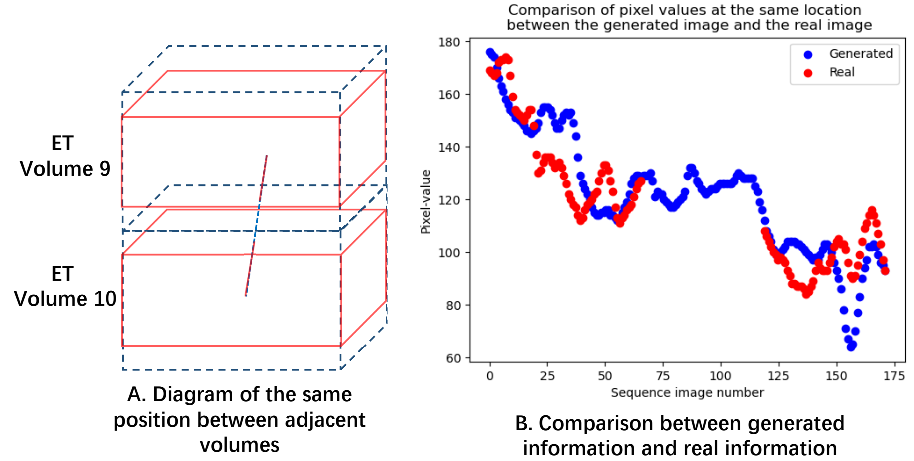

Figure S6 Schematic diagram of the comparison of pixel values at the same location between the generated image and the actual image, in adjacent volumes. The solid red line draws the actual data, and the dotted blue line draws the generated data in (A). (B) is the schematic diagram of the comparison of pixels at the straight line in (A).

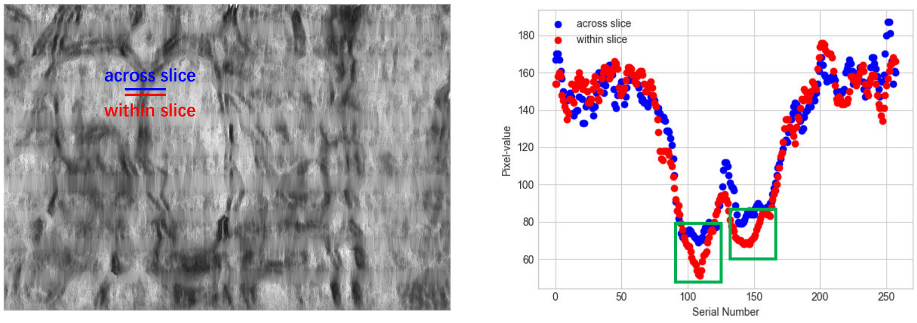

Figure S7 Schematic diagram of pixel variations across high-contrast feature boundaries across slices and within slices.

As shown in Fig. S7, the pixel variations across high-contrast feature boundaries across slices are not as sharp as those within slices, but the difference is small. This

indicates that the changing trend of the data we generate is consistent with the changing trend of the actual data. Meanwhile, this suggests that while the resolution across slices is slightly lower than within slices, the disparity is not significant.

In order to further quantify resolution ”within” and ”across” slices, we adopted the method based on image partial phase autocorrelation. We randomly selected 20 images in both the within section and the across section to calculate their resolution. The calculation results are shown in the Fig. S8. From the Fig. S8, it can be seen that the resolution of the ”within” and ”across” sections is basically the same. But from the median value, the resolution within the section is slightly better than that across the section.

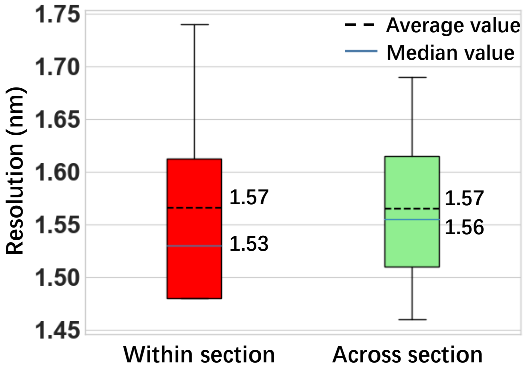

Figure S8 The boxplot of the resolution of different images within and across sections.
