# Supplementary material for: An intelligent workflow for sub-nanoscale 3D reconstruction of intact synapses from serial section electron tomography
Source: BMC Biol. 2023 Sep 25;21:198. doi: 10.1186/s12915-023-01696-x (PMC10519085; doi:10.1186/s12915-023-01696-x)
Supplement: Supplementary file 12 — Additional file 12: Text S4. Comparison of reconstruction results using different methods. The least squares method in TrakEM2 (TrakEM2-ls), Wang et al. proposed method [55] in TrakEM2 (TrakEM2-wang), elastic [56] in TrakEM2 (TrakEM2-elastic), ASAP [52] which used deep learning methods for image alignment, IMOD, and Irtool are used to compare with our workflow. [file 12915_2023_1696_MOESM12_ESM.pdf]

Supplementary file 12:

Comparison of reconstruction results using different methods

To demonstrate the superiority of our workflow, we compare it with the least squares method in TrakEM2 (TrakEM2-ls), Wang et al. proposed method in TrakEM2 (TrakEM2-wang), elastic in TrakEM2 (TrakEM2-elastic), ASAP which used deep learning methods for image alignment, IMOD, and Irtool. In Fig. S1, the box-plots of Structure Similarity Index Measure (SSIM) and Peak Signal-to-Noise Ratio (PSNR) of adjacent images between adjacent volumes aligned by different methods were used to show the alignment performance. It can be seen in the figure that the proposed workflow achieves better quantitative performance than others. Furthermore, Fig. S2 illustrated the reconstructed volumes by different methods. It can be seen in the figure of the partial enlarged image that the results obtained by our workflow are more continuous in the Z direction.

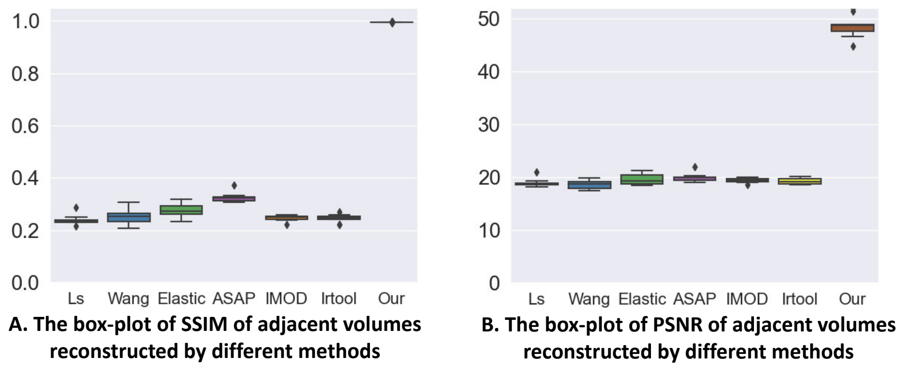

Figure S1 Comparison of SSIM and PSNR on adjacent surfaces of volumes aligned by different methods.

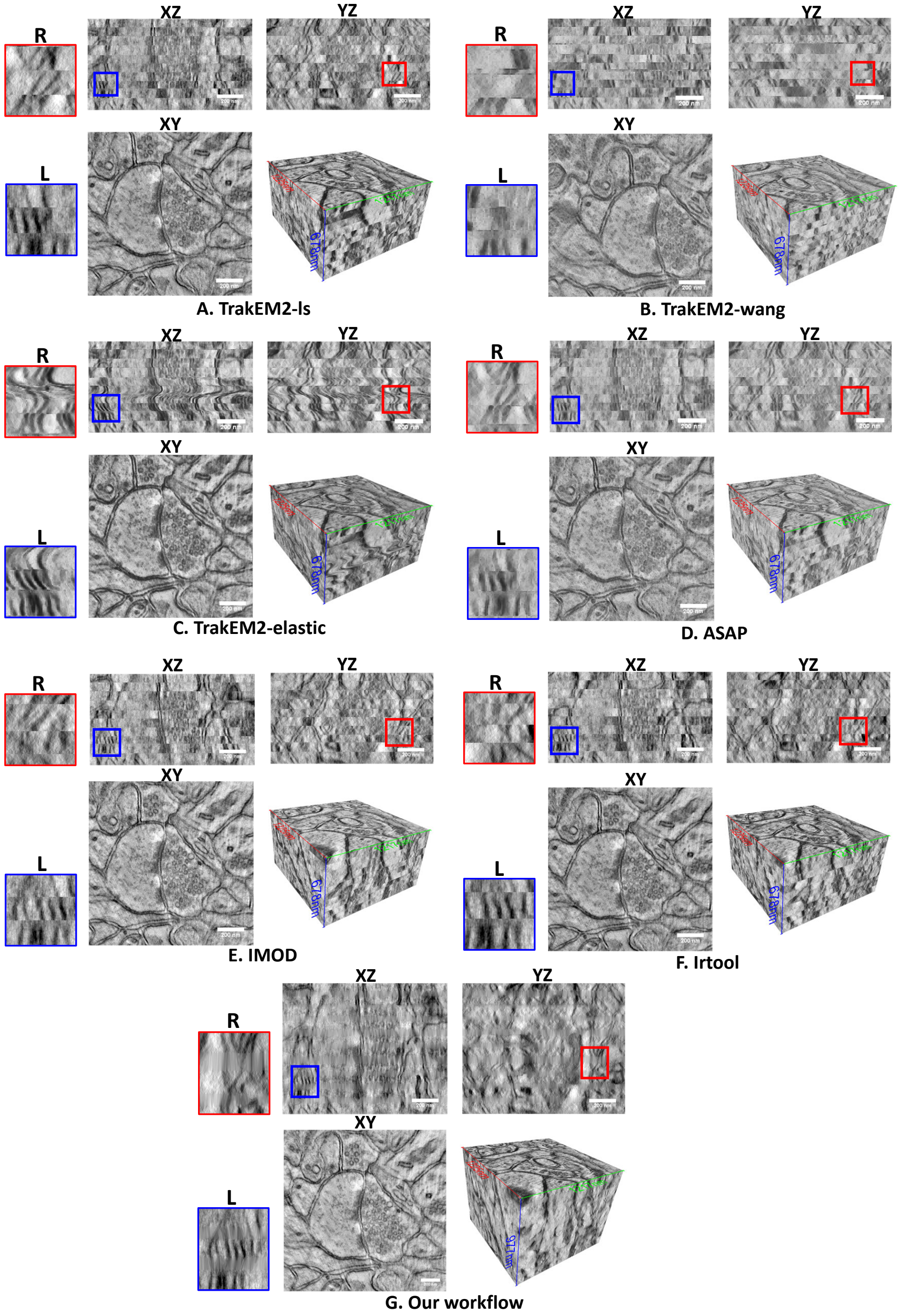

Figure S2 Comparison of the volumes reconstructed by different methods. The scale bar is 200 nm.
