# Supplementary material for: An intelligent workflow for sub-nanoscale 3D reconstruction of intact synapses from serial section electron tomography
Source: BMC Biol. 2023 Sep 25;21:198. doi: 10.1186/s12915-023-01696-x (PMC10519085; doi:10.1186/s12915-023-01696-x)
Supplement: Supplementary file 13 — Additional file 13: Text S5. Calculated lost thickness in different synaptic volumes. [file 12915_2023_1696_MOESM13_ESM.pdf]

## Supplementary file 13:

### Calculated lost thickness in different synaptic volumes

The calculated lost thickness in different synaptic volumes are shown in the Fig. S1 and Fig. S2. It reflects the overall material loss that has been taken into account in our analysis. The calculated average lost thickness in different volumes and sections is similar, as shown in the last row of Fig. S1 and the average lost thickness line of Fig. S2. However, there are noticeable differences in the lost thickness for each individual section. This variability may be attributed to different imaging conditions for each section, such as varying electron doses, astigmatism, and focus settings.

| Item(nm) | | Volume 1 | | Volume 2 | | Volume 3 | | Mean | |
|---|---|---|---|---|---|---|---|---|---|
| Section1 | Top | 0.00 | 10.62 | 21.91 | 29.22 | 13.94 | 35.19 | 11.95 | 25.01 |
| | bottom | 10.62 | | 7.30 | | 21.25 | | 13.06 | |
| Section2 | Top | 23.90 | 29.88 | 22.58 | 38.51 | 23.90 | 39.18 | 23.46 | 35.86 |
| | bottom | 5.98 | | 15.94 | | 15.27 | | 12.39 | |
| Section3 | Top | 27.89 | 43.82 | 15.94 | 33.20 | 38.51 | 39.18 | 27.45 | 38.73 |
| | bottom | 15.94 | | 17.26 | | 0.66 | | 11.29 | |
| Section4 | Top | 15.27 | 34.53 | 7.30 | 39.84 | 25.23 | 36.52 | 15.94 | 36.96 |
| | bottom | 19.26 | | 32.54 | | 11.29 | | 21.03 | |
| Section5 | Top | 27.22 | 35.19 | 1.33 | 26.56 | 30.54 | 31.21 | 19.70 | 30.99 |
| | bottom | 7.97 | | 25.23 | | 0.66 | | 11.29 | |
| Section6 | Top | 24.57 | 38.51 | 19.92 | 32.54 | 18.59 | 33.20 | 21.03 | 34.75 |
| | bottom | 13.94 | | 12.62 | | 14.61 | | 13.72 | |
| Section7 | Top | 25.90 | 26.56 | 21.91 | 33.20 | 10.62 | 35.86 | 19.48 | 31.87 |
| | bottom | 0.66 | | 11.29 | | 25.23 | | 12.39 | |
| Section8 | Top | 25.90 | 33.20 | 23.24 | 33.20 | 3.32 | 30.54 | 17.49 | 32.31 |
| | bottom | 7.30 | | 9.96 | | 27.22 | | 14.83 | |
| Section9 | Top | 13.28 | 33.86 | | | 13.94 | 23.90 | 13.61 | 28.88 |
| | bottom | 20.58 | | | | 9.96 | | 15.27 | |
| Section10 | Top | 13.94 | 38.51 | | | | | 13.94 | 38.51 |
| | bottom | 24.57 | | | | | | 24.57 | |
| Mean | | 16.23 | 32.47 | 16.64 | 33.28 | 16.93 | 33.86 | 16.69 | 33.39 |

Figure S1 Calculated lost thickness in different synaptic volumes and sections.

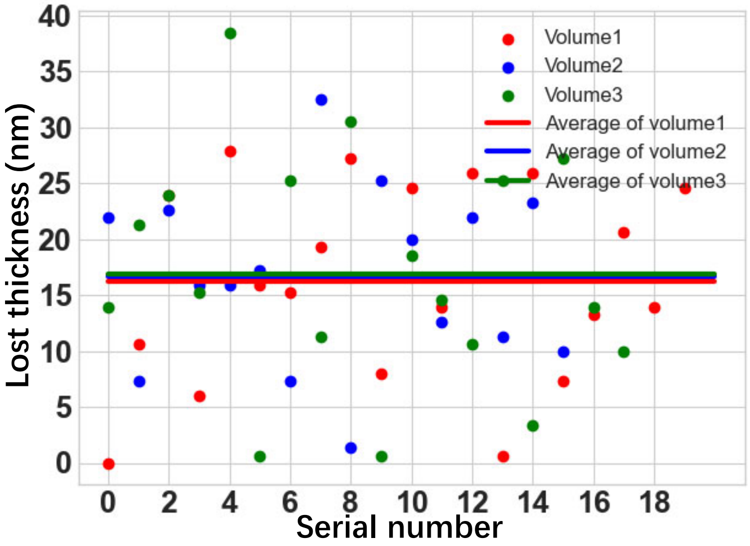


Figure S2 A plot of calculated lost thickness in different synaptic volumes and sections.
